# Halogenation engineered metal cluster assemblies

**DOI:** 10.1093/nsr/nwag154

**Published:** 2026-03-11

**Authors:** Xiao-Yan Shi, Xing-Nan Wang, Li-Xia Huang, Ya-Jie Wang, Zi-Ying Li, Hai-Yang Li, Zhen Han, Shuang-Quan Zang

**Affiliations:** Key Laboratory of Special Functional Molecular Materials (Zhengzhou University), Ministry of Education, Henan Key Laboratory of Crystalline Molecular Functional Materials, College of Chemistry, Zhengzhou University, Zhengzhou 450001, China; Key Laboratory of Special Functional Molecular Materials (Zhengzhou University), Ministry of Education, Henan Key Laboratory of Crystalline Molecular Functional Materials, College of Chemistry, Zhengzhou University, Zhengzhou 450001, China; Key Laboratory of Special Functional Molecular Materials (Zhengzhou University), Ministry of Education, Henan Key Laboratory of Crystalline Molecular Functional Materials, College of Chemistry, Zhengzhou University, Zhengzhou 450001, China; Key Laboratory of Special Functional Molecular Materials (Zhengzhou University), Ministry of Education, Henan Key Laboratory of Crystalline Molecular Functional Materials, College of Chemistry, Zhengzhou University, Zhengzhou 450001, China; Key Laboratory of Special Functional Molecular Materials (Zhengzhou University), Ministry of Education, Henan Key Laboratory of Crystalline Molecular Functional Materials, College of Chemistry, Zhengzhou University, Zhengzhou 450001, China; Key Laboratory of Special Functional Molecular Materials (Zhengzhou University), Ministry of Education, Henan Key Laboratory of Crystalline Molecular Functional Materials, College of Chemistry, Zhengzhou University, Zhengzhou 450001, China; Key Laboratory of Special Functional Molecular Materials (Zhengzhou University), Ministry of Education, Henan Key Laboratory of Crystalline Molecular Functional Materials, College of Chemistry, Zhengzhou University, Zhengzhou 450001, China; Key Laboratory of Special Functional Molecular Materials (Zhengzhou University), Ministry of Education, Henan Key Laboratory of Crystalline Molecular Functional Materials, College of Chemistry, Zhengzhou University, Zhengzhou 450001, China

**Keywords:** coinage metal clusters, self-assembly, supramolecular chirality, halogenation engineering, circularly polarized luminescence

## Abstract

Translating the elegant precision of molecular design into materials with predictable macroscopic functions represents a central goal in supramolecular and materials chemistry. Atomically precise coinage metal clusters offer an ideal platform for achieving this objective. However, progress is frequently frustrated by the limited and poorly tunable nature of the forces that direct assembly, a challenge that is particularly formidable in achieving the atomically precise transfer and amplification of chirality from the molecular to the supramolecular level. Herein, we introduce a halogenation engineering strategy that selectively installs distinct halogen atoms (F, Cl, Br) at the termini of peripheral ligands on a tetranuclear gold cluster to produce rationally tunable, directional noncovalent interactions, including hydrogen bonding, halogen bonding, and halogen···halogen interactions. This strategy transcends the inherent constraints of conventional noncovalent interactions in metal-cluster systems and significantly enriches the supramolecular toolbox. Leveraging this molecular-level control, we demonstrate that subtle modulation of the external solvent environment selectively biases the competition and cooperativity among these directional interactions, thereby enabling single-precursor self-assembly into multiple, structurally distinct, atomically precise polymorphs such as discrete monomers, finite helices, and infinite chains. Control is particularly evident in the formation of compactly twisted helical super-structures, in which molecular chirality is not only preserved but also efficiently transferred and amplified, resulting in dominant supramolecular chiroptical properties. This is exemplified by the freshly prepared crystals of (Au_4_-Br)_3_ trimer, which exhibits intense circularly polarized luminescence with a near-unity quantum yield (94%) and a high luminescence dissymmetry factor (*g*_lum_ = 0.04). This work establishes clear design principles for directing the complex and precise self-assembly of clusters via peripheral atom substitution, offering a rational methodology for the bottom-up fabrication of advanced chiral nanomaterials.

## Teaser

This study demonstrates a ‘halogenation engineering’ strategy that programs a single gold cluster into diverse helical superstructures, achieving both near-unity luminescence efficiency and exceptionally strong circularly polarized luminescence.

## INTRODUCTION

The fidelity with which molecular-level information is transduced into macroscopic structural complexity hinges critically on multivalent, anisotropic interactions originating from an atomically precise and photophysically active synthon [1–3]. Coinage metal clusters (CMCs), composed of Group IB elements and encapsulated by a protective organic ligand shell, exemplify this concept [4–6]. These clusters occupy a distinct size regime between atoms and nanoparticles, where quantum confinement effects are prominent, and exhibit discrete electronic energy levels along with tunable optical and catalytic properties [7,8]. Beyond their functional potential, the atomic precision of CMCs renders them ideal model systems for uncovering fundamental principles of self-assembly [9–11] and establishing well-defined structure–property relationships [12,13]. Despite considerable progress, achieving rational control over the assembly pathways of CMCs to yield precisely targeted topologies and functionalities remains a central challenge in supramolecular chemistry and nanoscience. Achieving such fine control is often hindered by the subtle and poorly tunable nature of the intrinsic forces that govern cluster organization [14]. This limitation is especially pronounced in the pursuit of supramolecular chirality, where the ultimate goal is not merely to preserve the chirality of the building block, but to amplify it through atomically precise and hierarchical assembly [15]. Among various strategies, chemical modification of the ligand shell represents a particularly effective approach for directing cluster assembly [16], prompting our exploration of halogenation engineering as a subtle yet powerful means of structural and functional control [17].

Halogenation engineering has been widely applied in organic crystal engineering [18–21], yet its potential for systematically directing the assembly of CMCs constitutes an emerging and promising frontier [22,23]. The efficacy of this approach arises from the unique electronic properties of halogen atoms [24,25]. When located at the ligand periphery of a cluster, highly polarizable halogens such as Cl and Br atoms exhibit an anisotropic electron distribution, leading to a region of positive electrostatic potential known as a *σ*-hole [26] on the halogen surface [27]. This feature enables the halogen to act as a highly directional Lewis acid. Incorporating this capability substantially expands the ‘supramolecular toolbox’ by enabling a variety of directional halogen-based interactions, including halogen bonding (a net attractive noncovalent interaction between the electrophilic region on a halogen atom and a nucleophilic site) [28] and halogen···halogen interactions (all interhalogen contacts, including the structurally diverse Type I–IV motifs that differ in geometry and directionality) [29], which can compete with and complement intrinsic inter-cluster interactions. This directional character is particularly valuable for inducing and amplifying supramolecular chirality [30–32]. Moreover, heavy halogens can induce strong spin-orbit coupling, thereby enhancing phosphorescence emission [33]. Despite these benefits, a systematic investigation into the synergistic influence of halogen choice on assembly structures, chiral expression, and resulting chiroptical properties in CMCs remains critically needed.

**Figure 1.**
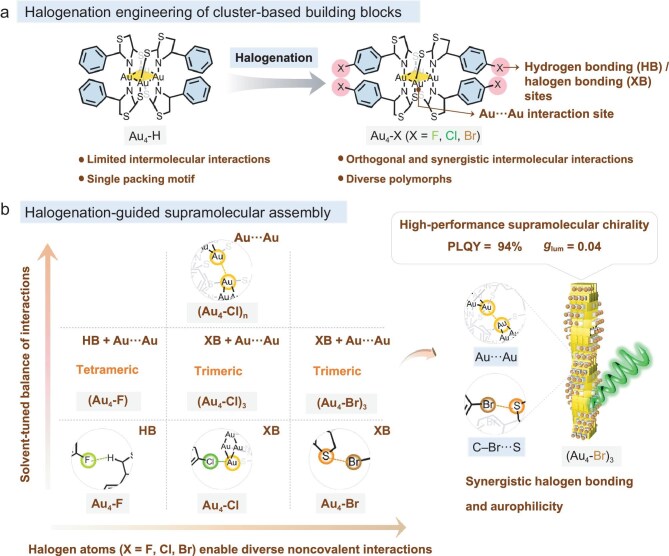
The halogenation engineering strategy for programmable supramolecular assembly. (a) Comparative analysis of assembly sites in Au_4_-H (non-halogenated) and its halogenated analogues Au_4_-X (X = F, Cl, Br). (b) Solvent-guided and selective assembly of the halogenated precursors into a family of distinct polymorphs, culminating in the high-performance chiroptical assembly of (Au_4_-Br)_3_.

Herein, we capitalize on this strategy by employing a tetranuclear gold (Au_4_) cluster protected by a thiazolethione-derived ligand as an ideal scaffold (Fig. 1a). Its inherent steric anisotropy and multiple peripheral interaction sites render it particularly suitable for investigating the influence of halogenation on the formation of diverse chiral architectures. The unmodified Au_4_‐H precursor exhibits limited intermolecular interaction diversity and structural homogeneity. The site-selective installation of halogen atoms (F, Cl, or Br) onto the peripheral ligands of the Au_4_ cluster not only introduces directional non-covalent interactions (including hydrogen bonding, halogen bonding, and halogen···halogen interactions) but also activates intrinsic Au···Au interactions between clusters in the orthogonal direction. We found that although all Au_4_-X clusters exist as monomers in solution, their solid-state assemblies exhibit striking divergence (Fig. 1b). This divergence arises from a synergistic interplay between Au···Au interactions and a rich array of newly introduced directional forces, including hydrogen bonding, halogen bonding, and halogen···halogen interactions. Moreover, solvent choice as an active, tunable parameter that dynamically modulates the balance between competing and cooperative non-covalent interactions, directs the clusters into helical stacking, thereby enabling chirality transfer and pronounced amplification. Using this approach, we successfully constructed a series of highly ordered crystalline architectures, ranging from discrete monomers to finite helical trimers and tetramers, as well as infinite one-dimensional chains. Through single crystal X-ray diffraction (SCXRD) and theoretical calculations, these atomically precise superstructures were fully resolved, enabling elucidation of their hierarchical assembly mechanisms and confirming halogen substitution as the key factor governing the final packing structures. This control is particularly evident in the formation of helical superstructures, where molecular chirality is efficiently transferred and amplified to the supramolecular level. A representative example is the chiral superstructure (Au_4_-Br)_3_, which adopts a helical arrangement driven by cooperative Au···Au interactions and C–Br···S halogen bonding, exhibiting bright circularly polarized luminescence (CPL) with a photoluminescence quantum yield (PLQY) of 94% and an exceptional luminescence asymmetry factor (*g*_lum_) of 0.04 (Fig. 1b). This work delineates the regulatory principles of halogenation engineering in cluster self-assembly, providing a strategic synthetic methodology for translating subtle molecular-level modifications into high-performance chiroptical materials.

## RESULTS AND DISCUSSION

The intrinsic atomic precision and three-dimensional modifiability of CMCs make them highly effective building blocks for the rational design and bottom-up construction of sophisticated supramolecular architectures. We specifically selected a tetranuclear gold (Au_4_) cluster scaffold, stabilized by thiazolethione ligands, as the foundational building block [34]. This choice was guided by two key considerations. First, the terminal position of the ligand framework is amenable to ‘halogenation engineering’ (Figs S1‒S16), which allows for the systematic incorporation of various halogen atoms (F, Cl, Br) to investigate how such peripheral atomic modifications influence the resulting supramolecular packing and the development of pronounced chiroptical activity. Second, this cluster inherently exhibits a spatially distributed array of weak interaction sites across multiple vectors, a structural characteristic that facilitates the modulation of intermolecular forces and the controlled assembly of crystalline architectures.

**Figure 2.**
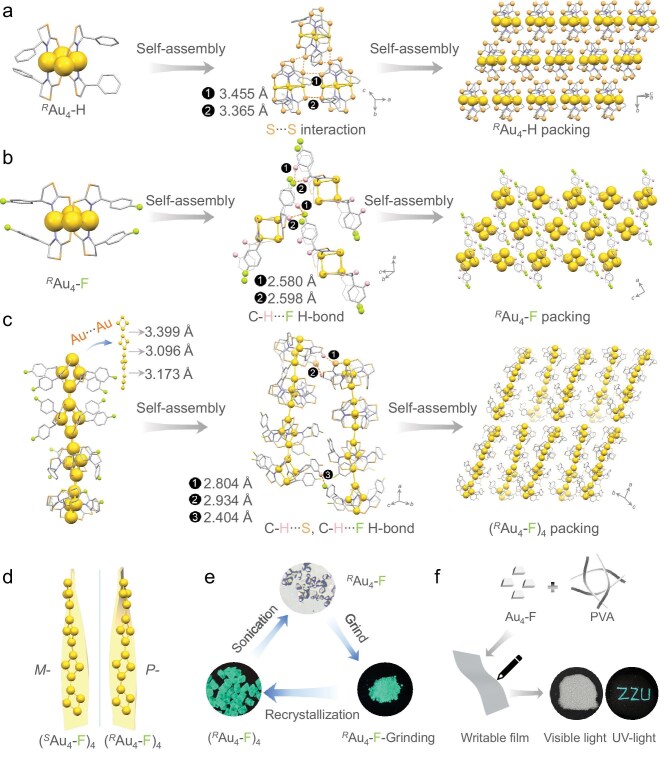
Crystal structures and photoluminescence properties of *^R^*Au_4_-H, *^R^*Au_4_-F, and (*^R^*Au_4_-F)_4_. Intermolecular interactions and molecular stacking based on the single-crystal structures of (a) *^R^*Au_4_-H, (b) *^R^*Au_4_-F, and (c) (*^R^*Au_4_-F)_4_. Color code: Au, golden yellow; C, dark gray; S, orange yellow; F, light green; N, blue. Hydrogen atoms are omitted for clarity. (d) The helical metallic cores within the (*^S^*Au_4_-F)_4_ and (*^R^*Au_4_-F)_4_ clusters. (e) Schematic transformation pathways among *^R^*Au_4_-F, *^R^*Au_4_-F-Grinding, and (*^R^*Au_4_-F)_4_. (f) Preparation process of writable *^R^*Au_4_-F cluster-based film.

The solid-state self-assembly behavior of the halogen-free (H-terminated) parent cluster Au_4_-H was characterized using X-ray crystallography, as shown in Fig. 2a, *^R^*Au_4_-H crystallizes in the orthorhombic chiral space group *P*2_1_2_1_2_1_. The *^R^*Au_4_-H molecule consists of a central rhombic Au_4_ core surrounded by four chiral ligands, which bind in a bidentate manner via their S and N atoms. This coordination mode results in the Au atoms being partially exposed, potentially serving as active sites for aurophilic interactions. Moreover, the exposed S atoms and terminal benzene rings of the ligands are also expected to play a role in supramolecular assembly (Fig. S17). Further analysis of the intermolecular weak interactions reveals that the *^R^*Au_4_-H crystal exhibits S···S interactions [35] in three-dimensional space (Fig. S18), leading to a layered stacking arrangement along the *b* axis. The intermolecular Au···Au distance is 3.559 Å (Fig. S19), slightly larger than the sum of the van der Waals radii of Au atoms (3.32 Å), indicating the absence of significant Au···Au interactions [36]. The limited diversity of weak intermolecular interactions between the Au_4_-H clusters restricts the structural variability of its assembled architectures. Despite variations in crystallization conditions, the Au_4_‐H crystal consistently adopts a single packing motif. Powder X-ray diffraction (PXRD) analysis confirmed that the Au_4_‐H crystal exhibits high crystalline phase purity and excellent reproducibility (Fig. S20). In terms of photoluminescence properties, the Au_4_‐H crystal exhibits blue emission, with a maximum emission wavelength at 460 nm and a quantum yield of 7.9% (Fig. S21).

After introducing F atoms at the terminal position of the ligand, the non-luminescent Au_4_-F clusters were synthesized in a mixed solvent system of acetone and methanol. sc-XRD analysis revealed that *^R^*Au_4_-F crystallizes in the chiral space group *C*222_1_. The Au_4_ core features two complementary dihedral angles of 93.36° and 85.58°, both of which are close to 90°, indicating that the core structure is nearly square and possesses the longest diagonal distance (Fig. S22 and Table S1). This near-square arrangement of the Au_4_ cores suggests weakened or less differentiated Au···Au interactions in comparison to the luminescent rhombic analogues of Au_4_-H, which are typically characterized by stronger Au···Au interactions or more favorable ligand-to-metal charge transfer (LMCT) or ligand-to-metal-metal charge transfer (LMMCT) pathways [37,38]. This subtle geometric variation may significantly influence the electronic structure, thereby affecting both the characteristics of excited states and the efficiency of non-radiative decay processes. The influence of the halogenation engineering strategy on cluster packing is determined by the way halogen atoms participate in non-covalent interactions. Due to fluorine’s exceptionally high electronegativity and small atomic radius, its dense electron cloud inhibits *σ*-hole [39] formation, thereby restricting its packing influence primarily to hydrogen bonding [40]. Indeed, SCXRD analysis of*^R^*Au_4_-F revealed two distinct C–H···F interactions, with distances of 2.580 Å and 2.598 Å, and corresponding angles of 146.55° and 118.67°, respectively (Fig. 2b and Fig. S23). Both sets of values match typical hydrogen bonding features [41]. These hydrogen bonds promote the formation of a layered packing structure in *^R^*Au_4_-F (Fig. 2b). PXRD analysis further confirmed that this structure exhibits good crystallinity, phase purity, and reproducibility (Fig. S24). The self-assembly of Au_4_-F is predominantly driven by C–H···F hydrogen bonding, and that further modulation of assembly conditions may enable the engagement of alternative interaction sites, thereby leading to diverse packing structures.

Building on this, acetonitrile was selected as the primary crystallization solvent during single crystal growth, owing to its capacity as a hydrogen bonding acceptor, which potentially perturbs both intramolecular and intermolecular hydrogen bonding networks. Concurrently, dichloromethane was introduced as a kinetic modulator. Its rapid volatility drives the system toward a new thermodynamic steady state. Thereby a new crystalline phase, (Au_4_-F)_4_, was obtained by tuning the crystallization solvent of acetonitrile and dichloromethane (3:1, v/v) (Fig. 2c). SCXRD analysis reveals that (*^R^*Au_4_-F)_4_ crystallizes in the chiral space group *I*2. The fundamental unit is a tetrameric supramolecule in which four *ᴿ*Au_4_-F clusters are interconnected by aurophilic interactions [42] between their rhombic metallic cores (Fig. S25 and Table S2), with Au···Au distances of 3.399 Å, 3.096 Å, and 3.173 Å. These tetrameric (*^R^*Au_4_-F)_4_ assemblies further organize into a layered, array-like architecture along the *a* axis, stabilized by a cooperative network of C–H···S (2.804 Å and 2.934 Å) and C–H···F (2.404 Å) hydrogen bonds. Notably, a defining structural feature of these tetramers is their intrinsically helical metal core, which arises from sequential rotations of the four constituent Au_4_-F clusters and results in a cumulative twist of ∼90° from end to end. This atomic-level stereochemical control directly dictates the resulting supramolecular chiral structures. Despite identical packing motifs, the (*^S^*Au_4_-F)_4_ and (*^R^*Au_4_-F)_4_ enantiomers give rise to left-handed (*M*) and right-handed (*P*) helical architectures, respectively (Fig. 2d). PXRD analysis of the bulk crystalline sample confirms the phase purity and structural reproducibility of (*^R^*Au_4_-F)_4_ (Fig. S26). This well-defined and rigid supramolecular assembly gives rise to remarkable photophysical properties. In sharp contrast to the non-luminescent *^R^*Au_4_-F monomer, the crystalline (*^R^*Au_4_-F)_4_ assembly exhibits intense green luminescence with a PLQY of 20.68% (Fig. S27). A microsecond-scale emission lifetime confirms the phosphorescent character of this luminescence. This significant enhancement in emission can be attributed to the aurophilic interactions among the rhombic Au_4_ cores. To the best of our knowledge, such finite helical assemblies featuring a twisted metal core scaffold governed by aurophilic interactions remain exceptionally rare.

The facile and solvent-controlled access to two distinct polymorphs, the monomeric Au_4_-F and the tetrameric (Au_4_-F)_4_, prompted us to investigate the possibility of a structural transformation between them. High-resolution electrospray ionization mass spectrometry (ESI-MS) analysis of *^R^*Au_4_-F and (*^R^*Au_4_-F)_4_ revealed identical fragment peaks (Fig. S28), and their liquid-phase UV-Vis absorption spectra exhibited a high degree of similarity (Fig. S29). Together, these observations indicate that the supramolecular tetrameric structure of (*^R^*Au_4_-F)_4_ is a purely solid-state phenomenon, as it fully dissociates into the constituent *^R^*Au_4_-F monomers in solution. Mechanical grinding of the non-luminescent *ᴿ*Au_4_-F crystals yielded a green-emissive powder, designated as *ᴿ*Au_4_-F-Grinding, with a PLQY of 7.91% (Fig. S30). To elucidate the origin of this mechano-induced photoluminescence, PXRD analysis was conducted (Fig. S31). The diffraction pattern of the *ᴿ*Au_4_-F-Grinding sample closely resembles that of the parent *ᴿ*Au_4_-F crystals but shows a consistent shift of all peaks to higher 2*θ* values, indicating that mechanical stress induces lattice contraction. This reduction in interplanar spacing is responsible for activating green emission. Beyond its optical response, this ground material serves as a key intermediate in a fully reversible transformation cycle (Fig. 2e). It is noteworthy that recrystallization of the ground powder yields exclusively the (*^R^*Au_4_-F)_4_ crystal. Upon ultrasonic treatment or prolonged standing in the mother liquor, the green luminescent (*^R^*Au_4_-F)_4_ gradually converts to non-luminescent *^R^*Au_4_-F. This pronounced ‘turn-on’ mechanochromic luminescence [43], in which mechanical force induces the transformation of the non-emissive *^R^*Au_4_-F into a luminescent state, provides a direct pathway to practical applications. To demonstrate this potential, we fabricated a composite film by dispersing non-emissive *^R^*Au_4_-F crystals into a polyvinyl alcohol (PVA) matrix. As shown in Fig. 2f, the film functions as a writable, pressure-sensitive optical material. Applying localized pressure with a stylus generates a well-defined luminescent pattern that is invisible under ambient light but clearly visible upon UV irradiation. This demonstration highlights the potential of such halogenated gold clusters in advanced applications, including flexible displays, anti-counterfeiting technologies, and smart sensors.

Building upon the diverse structural transformations induced by F atom incorporation, we extended our investigation to systematically examine the Cl and Br substitution systems. In comparison to F, Cl and Br atoms exhibit lower electronegativity and greater electron density, which renders them more susceptible to *σ*-hole formation [31]. The positively charged *σ*-hole is capable of engaging with electron-rich sites within cluster molecules, thereby facilitating the formation of halogen bonding. As a result, it can be inferred that a greater diversity of intermolecular forces participate in the assembly process, which is expected to yield more advanced assembled structures with enhanced performance.

**Figure 3.**
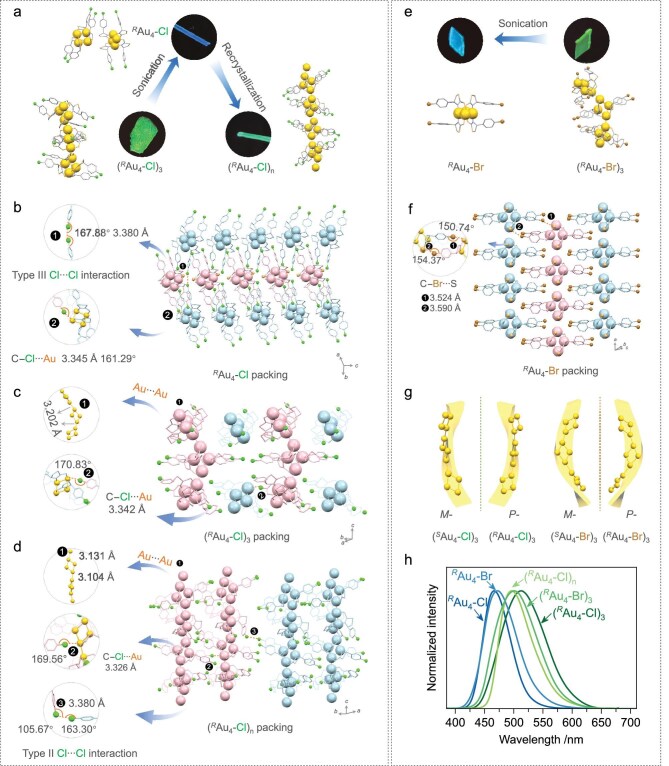
Crystal structures and photoluminescent properties of halogenated clusters Au_4_-Cl and Au_4_-Br series. (a) Single-crystal structures and transformation pathways among *^R^*Au_4_-Cl, (*^R^*Au_4_-Cl)_3_ and (*^R^*Au_4_-Cl)_n_, along with corresponding optical microscopy images of the crystals under UV light. Color code: Au, golden yellow; C, dark gray; S, orange yellow; N, blue; Cl, green. Hydrogen atoms are omitted for clarity. Interactions and molecular stacking based on the single-crystal structures of (b) *^R^*Au_4_-Cl, (c) (*^R^*Au_4_-Cl)_3_, and (d) (*^R^*Au_4_-Cl)_n_. (e) Single-crystal structures and transformation pathways between (*^R^*Au_4_-Br)_3_ and *^R^*Au_4_-Br, along with corresponding optical microscopy images of the crystals under UV light. (f) Interactions and molecular stacking in the single-crystal structure of *^R^*Au_4_-Br. (g) The helical metal cores in (*^R/S^*Au_4_-Cl)_3_ and (*^R/S^*Au_4_-Br)_3_. (h) Solid-state emission spectra of *^R^*Au_4_-Cl and *^R^*Au_4_-Br series crystals.

When a Cl atom is introduced at the terminal position of the benzene ring, three distinct crystal structures can be obtained by modulating the solvent type and polarity (Fig. 3a), with a clear transformation pathway observed among them (Fig. S32). The *^R^*Au_4_-Cl cluster crystallizes from a mixed solvent system of dichloromethane and acetonitrile at room temperature, adopting the orthorhombic chiral space group *P*2_1_2_1_2_1_. The asymmetric unit contains two crystallographically independent *ᴿ*Au_4_-Cl molecules (A and B), which differ primarily in their intramolecular noncovalent interactions (Fig. S33 and Table S3). Specifically, A exhibits a short Cl···Cl distance of 3.424 Å (∠C–Cl···Cl ≈ 80.76° and 116.92°), consistent with a Type Ⅳ Cl⋯Cl interaction [44,45]. In contrast, B lacks such intramolecular short contacts. SCXRD analysis further reveals that the packing of *^R^*Au_4_-Cl clusters is primarily governed by two distinct types of intermolecular weak interactions. One is a linear Cl···Cl interaction where both ∠C–Cl···Cl bond angles are 167.88°, falling within the range of *θ*_1_ ≈ *θ*_2_ ≈ 180°, consistent with Type Ⅲ Cl···Cl interactions [44]. The other is Cl···Au halogen bonding [46], wherein the Au atom exhibits metallic basicity and its nucleophilic region acts as a halogen bond acceptor, engaging the *σ*-hole on the terminal Cl atom. The Cl···Au distance is 3.345 Å, significantly shorter than the sum of their van der Waals radii (3.410 Å), and the bond angle ∠C–Cl···Au is 161.29°, nearly linear‐characteristic of halogen bonding [47]. The synergistic effect of these two interactions directs the *^R^*Au_4_-Cl clusters to form an ordered layered arrangement along the *b* axis (Fig. 3b). Photophysically, *ᴿ*Au_4_-Cl is a highly efficient blue emitter, exhibiting an emission peak at 465 nm and a remarkably high PLQY of 71.73%. This outstanding performance can be attributed to structural rigidification resulting from the synergistic effect of intramolecular and intermolecular Cl···Cl interactions and intermolecular Cl···Au halogen bonding, which can effectively suppress non-radiative decay pathways (Fig. S34).

The (*^R^*Au_4_-Cl)_3_ polymorph crystallizes in the monoclinic chiral space group *C*2, obtained from a mixed solvent system of acetone and acetonitrile (Fig. 3c, Fig. S35 and Table S4). The fundamental structural motif consists of a trimeric helical chain assembled from three *^R^*Au_4_-Cl clusters. These clusters are connected through strong inter-cluster Au···Au interactions, with a uniform distance of 3.202 Å between adjacent Au_4_ cores, shorter than the sum of their van der Waals radii (3.320 Å). The overall crystal packing is further stabilized by intermolecular Cl···Au halogen bonding. These interactions, with a distance of 3.342 Å and a nearly linear ∠C–Cl···Au angle of 170.83°, effectively bridge neighboring chains. Consequently, the cooperative action of Au···Au interactions and Cl···Au halogen bonding directs the formation of an ordered supramolecular architecture (Fig. 3c). This supramolecular assembly profoundly alters photophysical behavior. In contrast to the blue-emitting *ᴿ*Au_4_-Cl monomer, the (*ᴿ*Au_4_-Cl)_3_ polymorph exhibits a significantly red-shifted and intense green emission at 500 nm, with a PLQY of 36.24% (Fig. S36). This pronounced red shift is characteristic of aggregation and is directly attributed to the formation of inter-cluster Au···Au interactions, which give rise to new, lower-energy emissive states within the helical chain [48].

Furthermore, by changing the crystallization solvent to a *N,N*-dimethylformamide (DMF)/water system, a third polymorph, designated (*^R^*Au_4_-Cl)_n_, was isolated (Fig. 3d, Fig. S37 and Table S5). This phase crystallizes in the orthorhombic chiral space group *P*2_1_2_1_2_1_. SCXRD analysis reveals its defining structural feature which is the self-assembly of *ᴿ*Au_4_-Cl units into infinite one-dimensional (1D) chains through strong inter-cluster Au···Au interactions, with distances of 3.131 Å and 3.104 Å. These 1D chains are subsequently organized into a layered architecture via a synergistic network of halogen-based interactions. Crucially, a nearly linear C–Cl···Au halogen bonding (distance: 3.326 Å, ∠C–Cl···Au angle: 169.56°) [46] and a stabilizing Type II Cl···Cl interaction (distance: 3.380 Å), exhibiting the characteristic *L*-shaped geometry (∠C–Cl···Cl ≈ 105.67° and 163.30°) [45], act cooperatively to link adjacent chains along the *a* axis. The formation of extended 1D polymeric chains in (*ᴿ*Au_4_-Cl)_n_, governs its photophysical behavior, giving rise to a green emission at 500 nm characteristic of aurophilic interactions, with a PLQY of 10.06% (Fig. S38). This 1D chain constitutes the third distinct, atomically precise polymorph isolated to date, complementing the discrete monomer and finite trimer.

To elucidate the intrinsic origin of this structural diversity, systematic mass spectrometry and liquid-phase UV-Vis spectroscopic analyses were conducted on all three species (Figs S39 and S40), revealing the key insight that all three dissociate completely into the same monomeric *ᴿ*Au_4_-Cl unit in solution. This result indicates that, despite sharing a common solution-state precursor, their divergent supramolecular architectures emerge during crystallization through a synergistic interplay between solvent-mediated effects and intermolecular forces. PXRD analysis confirms high phase purity and reproducibility across all three polymorphs (Fig. S41). The ability to selectively isolate these distinct architectures, specifically a discrete monomer, a finite trimer, and an infinite chain, highlights the efficacy of Cl-for-H halogenation engineering in modulating supramolecular assembled processes for the rational design of complex cluster-based materials.

Based on the profound influence of chlorination on the assembly process, we extended our halogenation engineering strategy to bromine to systematically investigate how the increased atomic size and polarizability of the terminal halogen modulates the resulting supramolecular architectures. The Br atom is an ideal candidate for this role. Its high atomic number induces strong spin‐orbit coupling, and its polarizable electron cloud enables it to act as a potent *σ*-hole donor, a feature widely exploited in crystal engineering to direct intermolecular interactions [49]. Indeed, this strategy proved effective, yielding two distinct structural motifs, *ᴿ*Au_4_-Br and (*ᴿ*Au_4_-Br)_3_, which exhibit a well-defined, stimuli-responsive transformation pathway (Fig. 3e and Fig. S42).

The first polymorph, *^R^*Au_4_-Br, crystallizes in the *P*1 space group and is obtained from a mixed solvent system of dichloromethane and acetonitrile. The molecular packing of these crystals lack Au···Au interactions. Instead, it adopts a layered architecture that is stabilized by a synergistic network of intermolecular C–Br···S halogen bonding [47], which exhibit nearly linear angles of 150.74° and 154.37° with short contact distances of 3.524 Å and 3.590 Å, in addition to C–H···π interactions (Fig. 3f, Fig. S43 and Table S6). Photophysically, its properties are highly comparable to those of the *ᴿ*Au_4_-Cl analogue, showing an intense blue emission centered at 470 nm with an outstanding PLQY of 63.26% (Fig. 3h and Fig. S44).

In stark contrast, the second polymorph, (*^R^*Au_4_-Br)_3_, is selectively isolated from a mixture of dichloromethane and acetone (Fig. 3e). Although single crystals were obtained, significant ligand disorder prevented complete structural refinement by SCXRD. Therefore, its structure was determined through a combination of PXRD analysis and structural simulations, which confirmed phase purity and molecular packing (Fig. S45). This theoretical simulation and Pawley refinement reveal the defining structural feature, which is a helical assembly of three (*^R^*Au_4_-Br)_3_ cluster units via Au···Au interactions (Fig. S46). This aggregation induces a pronounced ∼30 nm bathochromic shift relative to the blue-emitting *ᴿ*Au_4_-Br monomer, resulting in a vibrant green emission centered at 500 nm and boosting the PLQY to an exceptional 94% (Fig. 3e, h, and Fig. S47). Crucially, EMI-MS and UV-Vis analyses confirm that both polymorphs dissociate into the same monomeric species in solution, demonstrating that the structural divergence arises from solvent-dependent aggregation (Figs S48 and S49). PXRD analysis confirms high phase purity and reproducibility for the two polymorphs (Fig. S50). Furthermore, the simulated structure of (*ᴿ*Au_4_-Br)_3_ is isomorphous with (*ᴿ*Au_4_-Cl)_3_. Both of them feature a helical metal core that is architecturally similar to that of the (*ᴿ*Au_4_-F)_4_ structure but significantly more compact and twisted (Figs 3g and 2d). A comparative analysis of the luminescence across the entire Au_4_-Cl and Au_4_-Br series of clusters confirms a consistent trend in which the formation of aggregated structures with enhanced Au···Au interactions systematically induces an emission redshift from blue to green (Fig. 3h).

Ultimately, these findings demonstrate that halogenation engineering is a powerful strategy for broadening the diversity of intermolecular interactions available to a cluster system. The introduction of a rich variety of halogen-based forces acting in concert with intrinsic aurophilicity effectively expands the supramolecular assembly toolbox. This enhanced control, guided by solvent choice, enables the precise construction of a diverse family of atomically precise CMC polymorphs ranging from discrete monomers to finite helices with rationally tunable photophysical properties.

**Figure 4.**
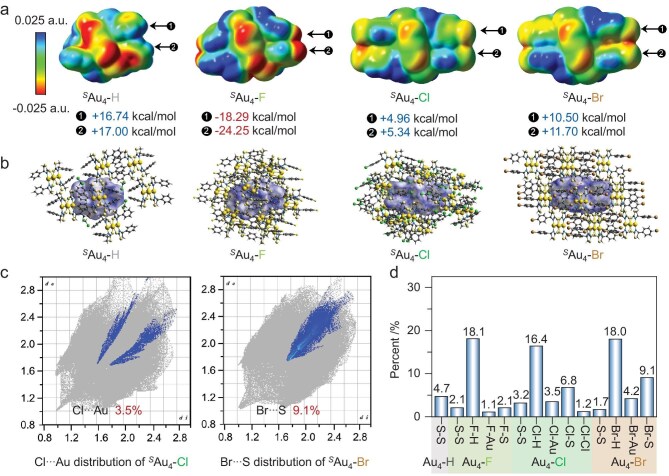
Computational studies of *^S^*Au_4_‐H and *^S^*Au_4_-X clusters. (a) ESP maps of different*^S^*Au_4_‐H and*^S^*Au_4_-X building units, highlighting the extremum ESP values on halogen atoms. (a.u., arbitrary units). (b) Hirshfeld surface of different building blocks. (c) 2D fingerprint plots of *^S^*Au_4_-Cl and *^S^*Au_4_-Br as examples. (d) Percentage contributions of selected close intermolecular contacts based on the Hirshfeld surface area.

To provide a clear theoretical rationale for the observed halogen-dependent assembly behavior, we conducted electrostatic potential (ESP) calculations and Hirshfeld surface analysis (HSA) on the series of cluster monomers. The ESP maps clearly illustrate the influence of the terminal halogen atom (Fig. 4a and Fig. S51). The highly electronegative F terminus exhibits a negative potential of −24.25 kcal/mol, while the Cl and Br termini display distinct regions of positive potential known as *σ*-holes. The magnitude of the *σ*-hole increases progressively from *^S^*Au_4_-Cl (+5.34 kcal/mol) to *^S^*Au_4_-Br (+11.70 kcal/mol). These computational findings offer a robust explanation for the experimentally observed packing structures. The electronegative F-terminus promotes the formation of C–H···F hydrogen bonds. In contrast, the Cl and Br atoms act as *σ*-hole donors and participate in directional halogen bonding interactions such as C–Cl···Au and C–Br···S and various types of Cl···Cl interactions, which also define the structural characteristics of their respective polymorph structures.

Hirshfeld surface analysis (HSA) of the single-crystal structures of the monomeric polymorphs offers additional quantitative evidence supporting these halogen-dependent trends. Visually, the total intermolecular contact area is substantially larger in the halogenated clusters than in the parent Au_4_-H and progressively increases with the halogen’s atomic number (F < Cl < Br), confirming an enrichment of non-covalent interactions (Fig. 4b). A quantitative decomposition of these interactions through two-dimensional (2D) fingerprint plots provides more detailed, atom-specific insights that are fully consistent with our experimental crystal structures (Fig. 4c, Figs S52–S55). The complete breakdown of contact percentages clearly illustrates the shifting balance of intermolecular forces (Fig. 4d). Relative to the halogenated series, the *^S^*Au_4_-H cluster exhibits the highest proportion of S···S contacts (4.7%), which is fully consistent with the experimental observation that its assembly is primarily driven by these interactions (Fig. 1a). Upon fluorination, the interaction landscape of *^S^*Au_4_-F becomes overwhelmingly dominated by F···H hydrogen bonds, which constitute a remarkable 18.1% of the total surface contact, again corroborating the crystal structure analysis. As we move to the heavier halogens, a clear shift in interaction preference is observed. The contribution from X···S contacts increases steadily from F (2.1%) to Cl (6.8%) to Br (9.1%), while the X···H contribution decreases. This trend underscores the increasing importance of halogen-based interactions in the chlorinated and brominated systems. Within the X···X halogen bonding category, only the *^S^*Au_4_-Cl cluster shows a discernible Cl···Cl contact, a finding that is also in perfect agreement with the single-crystal analysis. Overall, these theoretical analyses are in excellent agreement with the experimental data, confirming that our halogenation engineering strategy effectively enriches the range of weak intermolecular forces and provides a tunable means to direct the self-assembly of clusters into diverse and complex supramolecular architectures.

**Figure 5.**
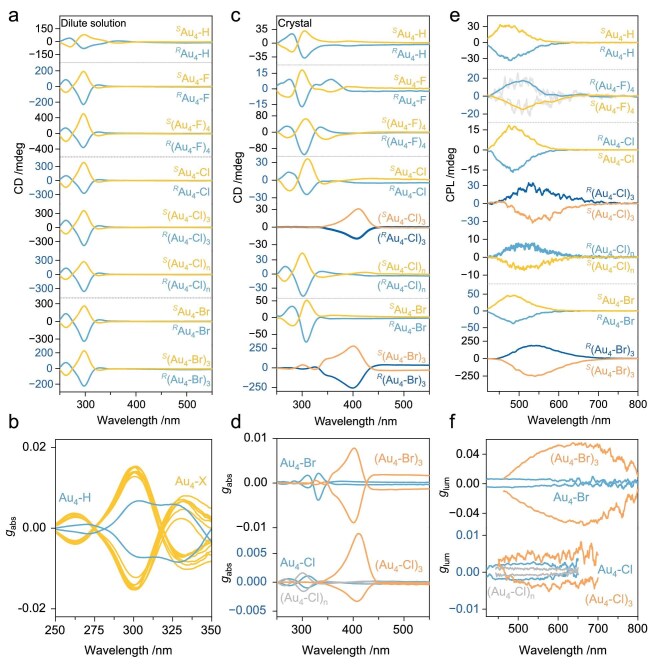
Chiroptical properties of *^R/S^*Au_4_-H and *^R/S^*Au_4_-X (X = F, Cl, Br) clusters and their assemblies. (a) CD spectra of *^R/S^*Au_4_-H and *^R/S^*Au_4_-X clusters and their assemblies in dilute solution (0.036 mM). (b) Corresponding *g*_abs_ values. (c) CD spectra of *^R/S^*Au_4_-H and *^R/S^*Au_4_-X clusters and their assemblies in the solid state. (d) *g*_abs_ values for different crystal structures in *^R/S^*Au_4_-Cl and *^R/S^*Au_4_-Br systems. (e) CPL spectra of *^R/S^*Au_4_-H and *^R/S^*Au_4_-X clusters and their assemblies in the solid state. (f) *g*_lum_ Values for different crystal structures in *^R/S^*Au_4_-Cl and *^R/S^*Au_4_-Br systems.

To investigate the chiroptical properties of the Au_4_-H and Au_4_-X series clusters and their assemblies, circular dichroism (CD) and circularly polarized luminescence (CPL) spectroscopy were performed in both solution and solid states. In dilute dichloromethane solutions, the CD spectra of all clusters exhibit remarkable similarity, as illustrated in Fig. 5a and Figs S56–S59. Both the parent *^R/S^*Au_4_-H and halogenated *^R/S^*Au_4_-X clusters display intense, exciton-coupled Cotton effects within the 250–320 nm range, which corresponds to ligand-centered absorptions. This observation indicates that significant intramolecular interactions between aromatic ligand groups persist even in solution, thereby establishing well-defined intrinsic molecular chirality. The nearly superimposable CD spectra of the halogenated derivatives further demonstrate that the nature of the terminal halogen atom exerts negligible influence on the ground-state chiral configuration in solution, confirming that all species exist predominantly as discrete monomers. A systematic analysis of the absorption dissymmetry factors (*g*_abs_) reveals that the halogenated clusters consistently exhibit slightly enhanced *g*_abs_ values relative to Au_4_-H, indicating that halogenation marginally amplifies the intrinsic chirality of the isolated monomeric units (Fig. 5b).

In the crystalline state, the CD spectra are closely correlated with the supramolecular packing arrangements as shown in Fig. 5c and Figs S60–S63. Most polymorphs including *^R/S^*Au_4_-F, (*^R/S^*Au_4_-F)_4_, *^R/S^*Au_4_-Cl, (*^R/S^*Au_4_-Cl)_n_, and *^R/S^*Au_4_-Br exhibit Cotton effects predominantly in the 250–320 nm range indicating that their solid-state chirality is primarily governed by the intrinsic molecular chirality of the constituent clusters. A notable exception is observed in the helically assembled (*^R/S^*Au_4_-Cl)_3_ and (*^R/S^*Au_4_-Br)_3_ trimers, which display intense Cotton effects near 400 nm, a spectral region corresponding to metal core absorption. This feature represents a clear signature of supramolecular chirality originating from the helical arrangement of the metal cores. Notably although the (*^R/S^*Au_4_-F)_4_ tetramer also possesses a helical core, its structural twist is significantly less pronounced, resulting in no dominant supramolecular chirality contribution to its CD spectrum. This contrast highlights the effective and efficient transfer of chirality from the molecular to the supramolecular level, particularly in the Cl- and Br-based trimeric assemblies. To the best of our knowledge, CMC platforms that enable unambiguous, atomic-level resolution of chirality transfer across length scales remain exceptionally rare. The extent of chiral amplification is quantified by the *g*_abs_ values shown in Fig. 5d. The helical (*^R/S^*Au_4_-Cl)_3_ and (*^R/S^*Au_4_-Br)_3_ structures exhibit substantially enhanced *g*_abs_ values compared to their respective monomers, with (*^R/S^*Au_4_-Br)_3_ achieving a notably high *g*_abs_ of 0.01. Such a value is exceptionally rare among crystalline cluster assemblies, highlighting the effectiveness of the helical architecture in amplifying molecular chirality.

As the cluster monomers are non-luminescent in solution, CPL measurements were conducted on crystalline solid samples (Fig. 5e and Figs S64–S67). The emissive monomeric polymorphs of *^R/S^*Au_4_-H, *^R/S^*Au_4_-Cl and *^R/S^*Au_4_-Br exhibit mirror-symmetric CPL signals that align with their blue luminescence. For the aggregated structures, the CPL signals are red-shifted, consistent with their green emission. Notably, the helically stacked (*^R/S^*Au_4_-Cl)_3_ and (*^R/S^*Au_4_-Br)_3_ trimers display the most pronounced red-shift, with signals centered near 540 nm. This finding further supports the effective transfer of chirality from the monomeric units to the emissive supramolecular state. The *g*_lum_ values provide a definitive confirmation of this chiral amplification (Fig. 5f). The *g*_lum_ value for (*^R/S^*Au_4_-Cl)_3_ is measured at 0.005, while that for (*^R/S^*Au_4_-Br)_3_ reaches a remarkable 0.04, which is among the highest values reported for crystalline materials. This comprehensive chiroptical analysis offers a demonstration of our halogenation engineering strategy. It not only enables the formation of diverse crystalline assembled architectures through the introduction of competing intermolecular forces, but also, by leveraging directional halogen bonding, establishes a robust pathway for the efficient transfer and amplification of chirality from the molecular to the supramolecular level, thereby providing a systematic and atomically precise illustration of structure‐property relationships in chiral nanomaterials.

## CONCLUSION

In summary, this work establishes halogenation engineering as a powerful and versatile strategy for achieving precise control over the self-assembly behavior, luminescent properties, and chiroptical performance of Au_4_ clusters. Systematic replacement of terminal hydrogen atoms with halogens introduces a tunable set of directional interaction sites across the cluster building blocks. This fine-tunes the delicate interplay between intrinsic Au···Au interactions and multiple halogen-mediated noncovalent forces, including hydrogen bonding, halogen bonding, and halogen···halogen interactions. By rationally selecting solvents to modulate the relative strengths of these interactions, we successfully directed the hierarchical assembly of a single Au_4_ precursor into a structurally diverse and functionally distinct family of supramolecular architectures, thereby elucidating their underlying assembly pathways. The most striking outcome is observed in the (*^R^*Au_4_‐Br)_3_ assembly, where cooperative aurophilic interactions and directional C–Br···S halogen bonding jointly drive the formation of a compact helical superstructure. This architecture facilitates highly efficient chirality transfer and amplification from the molecular to the supramolecular level, yielding an exceptional quantum yield of 94% and a remarkably high *g*_lum_ of 0.04. These high-performance crystalline materials hold significant promise for applications in chiral photonics and stimuli-responsive functional materials. This work establishes a robust experimental and theoretical foundation for halogen engineering in Au(I) systems. Halogenation engineering exhibits broad applicability across diverse d^10^ metal systems. However, its successful adaptation to distinct d^10^ metal platforms requires systematic tuning of the synergistic interplay between the metal center’s intrinsic electronic structure and halogen-mediated non-covalent interactions, thereby enabling precise control over supramolecular assembly behavior. Addressing this fundamental challenge represents a central focus of our future work on cluster-based supramolecular assembly materials.

## Supplementary Material

nwag154_Supplemental_Files
