# Supplementary material for: Halogenation engineered metal cluster assemblies
Source: Natl Sci Rev. 2026 Mar 11;13(10):nwag154. doi: 10.1093/nsr/nwag154 (PMC13245388; doi:10.1093/nsr/nwag154)
Supplement: nwag154_Supplemental_Files [file nwag154_supplemental_files.zip › Revised SupportingInformation-0301.pdf]

Supplementary data

Halogenation Engineered Metal Cluster Assemblies

Xiao-Yan Shi, Xing-Nan Wang, Li-Xia Huang, Ya-Jie Wang*, Zi-Ying Li, Hai-Yang Li, Zhen Han* and Shuang-Quan Zang*

Key Laboratory of Special Functional Molecular Materials (Zhengzhou University), Ministry of Education, Henan Key Laboratory of Crystalline Molecular Functional Materials, College of Chemistry, Zhengzhou University, Zhengzhou 450001, P. R. China.

* Correspondence: wangyajie92@zzu.edu.cn

* Correspondence: hanzhen77@zzu.edu.cn

* Correspondence: zangsqzg@zzu.edu.cn

This file includes:
Supplementary Note 1-4
Supplementary Figures S1 to S67
Supplementary Tables S1 to S6
References

Supplementary Note 1

Materials

All starting materials and solvents were purchased from commercial suppliers and used without further purification. Specifically, (R)-2-Amino-2-(4-fluorophenyl)ethan-1-ol hydrochloride (^{R}F), (S)-2-Amino-2-(4-fluorophenyl)ethan-1-ol hydrochloride (^{S}F), (R)-2-Amino-2-(4-chlorophenyl)ethan-1-ol hydrochloride (^{R}Cl), and (S)-2-Amino-2-(4-chlorophenyl)ethan-1-ol (^{S}Cl) hydrochloride were obtained from Shanghai Macklin Biochemical Co., Ltd. (R)-2-Amino-2-(4-bromophenyl)ethanol (^{R}Br) and (S)-2-Amino-2-(4-bromophenyl)ethanol (^{S}Br) were purchased from Shanghai Energy Chemical Co., Ltd. Carbon disulfide solution was supplied by Beijing Leyan Technology Co., Ltd. Poly(vinyl alcohol) 1799 (PVA) was procured from Aladdin Reagent Co., Ltd. (Shanghai, China).

Supplementary Note 2

Synthesis

Synthesis of ligands

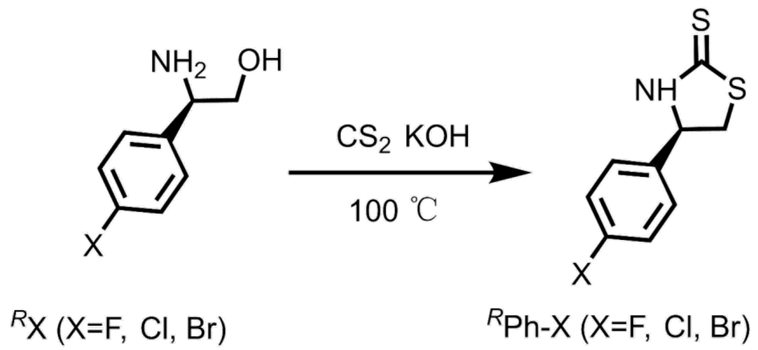

Scheme S1. The synthetic route of Ph-X ligand.

We used the synthesis procedure of ^{R}Ph-F as an illustrative example.

$^{\boldsymbol{R}}$**Ph-F** was synthesized by modifying a previously reported method[1]. In detail, ^{R}F (1 g, 6.445 mmol) was dissolved in 34 mL of 1 mol/L aqueous KOH solution, followed by the addition of carbon disulfide (50 mmol, 2.265 mL). The resulting mixture was stirred at 100 °C for 16 hours under continuous monitoring. Upon completion of the reaction, as indicated by thin-layer chromatography (TLC), the mixture was cooled to room temperature. The reaction mixture was then extracted twice with 50 mL of dichloromethane (DCM), and the organic layer was dried over anhydrous sodium sulfate (Na_2SO_4). The crude product was purified by flash column chromatography using DCM as the eluent. The final product was obtained as white crystalline solids.

The $^{\boldsymbol{R/S}}$**Ph-H** ligand was synthesized following the previously reported procedure.1

$^{\boldsymbol{R}}$**Ph-F** :^{1}H NMR (600 MHz, DMSO-d_6): δ 10.55 (s, 1H, N*H*), 7.43-7.41 (d, *J* = 12 Hz, 2H, H_{arom}), 7.27-7.24(d, *J* = 18 Hz, 2H, H_{arom}), 5.41-5.39 (dd, *J* = 8.3, 6.2Hz, 1H, SCH_2CH), 3.99-3.95 (dd, *J* = 11.4, 8.3 Hz, 1H, SCH_2CH), 3.37-3.35(dd, *J* = 11.4, 6.2 Hz, 1H, HNC*H*Ph). ^{13}C NMR (151 MHz, DMSO-d_6): δ 199.64, 163.20, 161.58, 136.43, 136.41, 128.85, 128.79, 116.09, 115.94, 66.19, 41.06. Yield: 70.03%.

$^{\boldsymbol{S}}$**Ph-F**: ^{1}H NMR (600 MHz, DMSO-d_6): δ 10.55 (s, 1H, N*H*), 7.43-7.41 (d, *J* =

12 Hz, 2H, H_{arom}), 7.27-7.24(d, J = 18 Hz, 2H, H_{arom}), 5.41-5.39(dd, J = 8.3, 6.2 Hz, 1H, SCH_2CH), 3.99-3.95 (dd, J = 11.4, 8.3 Hz, 1H, SCH_2CH), 3.37-3.35(dd, J = 11.4, 6.2 Hz, 1H, HNCHPh). ^{13}C NMR (151 MHz, DMSO-d_6): δ 199.64, 163.20, 161.58, 136.43, 136.41, 128.85, 128.79, 116.09, 115.94, 66.19, 41.06. Yield: 49.88%.

^{R}Ph-Cl: ^{1}H NMR (600 MHz, DMSO-d_6): δ 10.57 (s, 1H, NH), 7.50-7.48 (d, J = 12 Hz, 2H, H_{arom}), 7.40-7.39(d, J = 6 Hz, 2H, H_{arom}), 5.42-5.39 (dd, J = 8.4, 6.0 Hz, 1H, SCH_2CH), 4.00-3.97 (dd, J = 11.4, 8.4Hz, 1H, SCH_2CH), 3.37-3.34 (dd, J = 11.4, 6.0 Hz, 1H, HNCHPh). ^{13}C NMR (151 MHz, DMSO-d_6): δ 199.80, 139.25, 133.28, 129.19, 128.59, 66.12, 40.95. Yield: 64.76%.

^{S}Ph-Cl: ^{1}H NMR (600 MHz, DMSO-d_6): δ 10.57 (s, 1H, NH), 7.50-7.48 (d, J = 12 Hz, 2H, H_{arom}), 7.40-7.39(d, J = 6 Hz, 2H, H_{arom}), 5.42-5.39 (dd, J = 8.3, 6.1 Hz, 1H, SCH_2CH), 4.00-3.97 (m, J = 11.4, 8.4 Hz, 1H, SCH_2CH), 3.37-3.34 (m, J = 11.4, 6.0 Hz, 1H, HNCHPh). ^{13}C NMR (151 MHz, DMSO-d_6): δ 199.80, 139.25, 133.28, 129.19, 128.60, 66.12, 40.94. Yield: 64.76%.

^{R}Ph-Br: ^{1}H NMR (600 MHz, DMSO-d_6): δ 10.56 (s, 1H, NH), 7.63-7.62 (d, J = 6 Hz, 2H, H_{arom}), 7.34-7.32 (d, J =12 Hz, 2H, H_{arom}), 5.40-5.38 (dd, J = 8.4, 6.0 Hz, 1H, SCH_2CH), 4.00-3.97 (dd, J = 11.4, 8.4 Hz, 1H, SCH_2CH), 3.36-3.34 (dd, J = 11.4, 6.0 Hz, 1H, HNCHPh). ^{13}C NMR (151 MHz, DMSO-d_6): δ 199.82, 139.68, 132.12, 128.91, 121.81, 66.17, 40.88. Yield: 60.04%.

^{S}Ph-Br: ^{1}H NMR (600 MHz, DMSO-d_6): δ 10.56 (s, 1H, NH), 7.63-7.62 (d, J = 6 Hz, 2H, H_{arom}), 7.34-7.32 (d, J =12 Hz, 2H, H_{arom}), 5.40-5.38 (dd, J = 8.4, 6.0 Hz, 1H, SCH_2CH), 4.00-3.97 (dd, J = 11.4, 8.4 Hz, 1H, SCH_2CH), 3.36-3.33 (dd, J = 11.4, 6.0 Hz, 1H, HNCHPh). ^{13}C NMR (151 MHz, DMSO-d_6): δ 199.82, 139.68, 132.12, 128.92, 121.81, 66.17, 40.88. Yield: 60.04%.

Synthesis of clusters

***R*Au$_4$-X (X=F, Cl, Br)** clusters are used as an example.

***R*Au$_4$-H** was synthesized following the previously reported procedure.1

***R*Au$_4$-F:** ^{R}Ph-F (4.26 mg, 0.02 mmol) was dissolved in 1 mL of methanol, and Me_2SAuCl (6 mg, 0.02 mmol) was dissolved in 1 mL of acetone. The two solutions were thoroughly mixed, followed by the addition of 10 μL of triethylamine. Block-shaped crystals formed after 48 hours of standing.

(^{R}Au$_4$-F)$_4$: ^{R}Ph-F (4.26 mg, 0.02 mmol) was dissolved in 1 mL of acetonitrile, and Me_2SAuCl (6 mg, 0.02 mmol) was dissolved in 3 mL of dichloromethane (DCM). The two solutions were thoroughly mixed, followed by the addition of 10 μL of triethylamine. Block-like crystal was obtained after the mixture was allowed to stand for 48 hours.

***R*Au$_4$-Cl:** ^{R}Ph-Cl (4.60 mg, 0.02 mmol) was dissolved in 1 mL of acetonitrile, and Me_2SAuCl (6 mg, 0.02 mmol) was dissolved in 3 mL of DCM. The two solutions were thoroughly mixed, followed by the addition of 10 μL of triethylamine. Needle-like crystal was obtained after the mixture was allowed to stand for 48 hours.

(^{R}Au$_4$-Cl)$_3$: ^{R}Ph-Cl (4.26 mg, 0.02 mmol) was dissolved in 1 mL of acetonitrile, and Me_2SAuCl (6 mg, 0.02 mmol) was dissolved in 3 mL of acetone. The two solutions were thoroughly mixed, followed by the addition of 10 μL of triethylamine. Sheet-like crystals were obtained after the mixture was allowed to stand for 12 hours.

(^{R}Au$_4$-Cl)$_n$: A 900 μL of 1 mM Au_4-Cl in N, N-Dimethylformamide (DMF) solution was transferred to a centrifuge tube, followed by the addition of 100 μL of deionized water. Needle-like crystals formed after the mixture was allowed to stand for 24 hours.

***R*Au$_4$-Br:** ^{R}Ph-Br (5.46 mg, 0.02 mmol) was dissolved in 1 mL of acetonitrile, and Me_2SAuCl (6 mg, 0.02 mmol) was dissolved in 3 mL of DCM. The two solutions were mixed thoroughly, followed by the addition of 10 μL of triethylamine. Sheet-like crystals were obtained after the mixture was allowed to stand for 10 hours.

(^{R}Au$_4$-Br)$_3$: ^{R}Ph-Br (5.46 mg, 0.02 mmol) was dissolved in 1 mL of DCM, and

Me_2SAuCl (6 mg, 0.02 mmol) was dissolved in 1 mL of acetone. The two solutions were mixed thoroughly, followed by the addition of 10 μL of triethylamine. Sheet-like crystals were obtained after the mixture was allowed to stand for 30 minutes.

Supplementary Note 3

Preparation of writable Au_4-F cluster-based films

20 mg of $^{R}Au_4$-F clusters were uniformly dispersed in 1 mL of a 50 mg/mL aqueous solution of polyvinyl alcohol. A 200 μL aliquot of the resulting homogeneous mixture was drop-cast onto a 2×2 cm^2 glass slide and spin-coated to form a uniform circular thin film. The sample was subsequently dried in an oven at 80 °C, yielding a composite film that exhibits pronounced mechanoresponsive luminescence upon application of pressure.

Supplementary Note 4

Characterizations

Unless otherwise specified, all characterizations were performed at room temperature. X-ray diffraction (XRD) patterns were recorded using a Rigaku MiniFlex diffractometer equipped with Cu-*K*α radiation (λ = 1.54178 Å). Proton nuclear magnetic resonance (^{1}H NMR) and carbon-13 nuclear magnetic resonance (^{13}C NMR) spectra were acquired on a Bruker AVANCE 600 MHz spectrometer. Circular dichroism (CD) spectra were measured using a JASCO J-1500 spectropolarimeter. Circularly polarized luminescence (CPL) spectra were collected on a JASCO CPL-300 spectrometer. Electrospray ionization mass spectrometry (ESI-MS) was conducted on an AB SCIEX X500R Q-TOF spectrometer. Steady-state photoluminescence (PL) spectra were obtained using a HORIBA FluoroLog-3 fluorescence spectrometer. Luminescence lifetimes were measured using the same instrument in time-correlated single-photon counting (TCSPC) mode. UV-Visible absorption spectra at room temperature was recorded on a UV-1900 Shimadzu spectrophotometer. Single crystal X-ray diffraction (SCXRD) measurements were performed on a Bruker D8 venture diffractometer with Mo-K*α* radiation (λ = 0.71073 Å). Data collection and reduction were processed using the $CrysAlis^{Pro}$ software suite. The structure was solved using intrinsic phasing methods (SHELXT-2015)[2] and refined by full-matrix least squares on F^2 using OLEX2[3], which utilizes the SHELXL-2018/3 module[4,5]. Detailed information of the SCXRD data, intensity collection procedure, and refinement results of these crystals are summarized in Table S1-S6. The $(^{R}Au_4\text{-}Br)_3$ structural model was constructed using the Materials Studio software suite. Geometric optimization was subsequently performed using the Forcite module with the Universal Force Field and Ewald summation method to ensure accurate structural refinement. Following optimization, the Powder X-Ray Diffraction (PXRD) pattern was calculated using the Reflex Plus module. Finally, Pawley refinement was applied to fine-tune the simulated PXRD profile, resulting in the refined pattern and the corresponding R_{wp} and R_p values, which serve as quantitative

indicators of the goodness of fit.

Supplementary figures

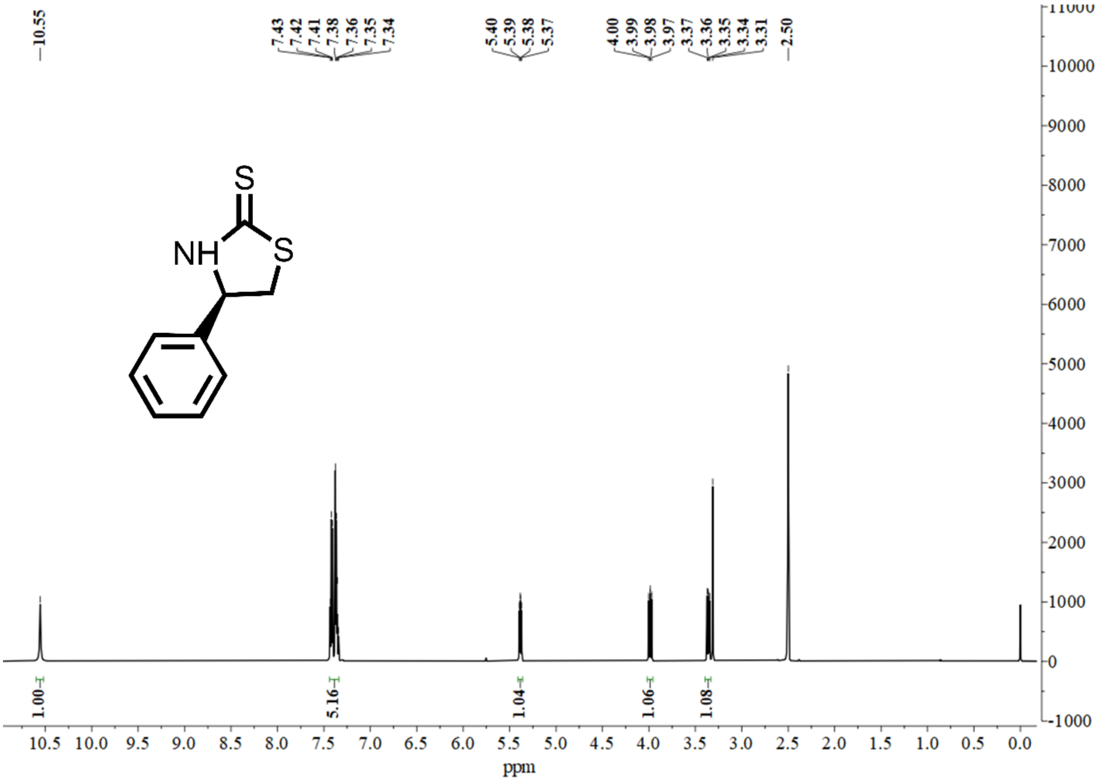

Figure S1. ^{1}H NMR spectrum of ^{R}Ph-H (600 MHz, DMSO-d_6).

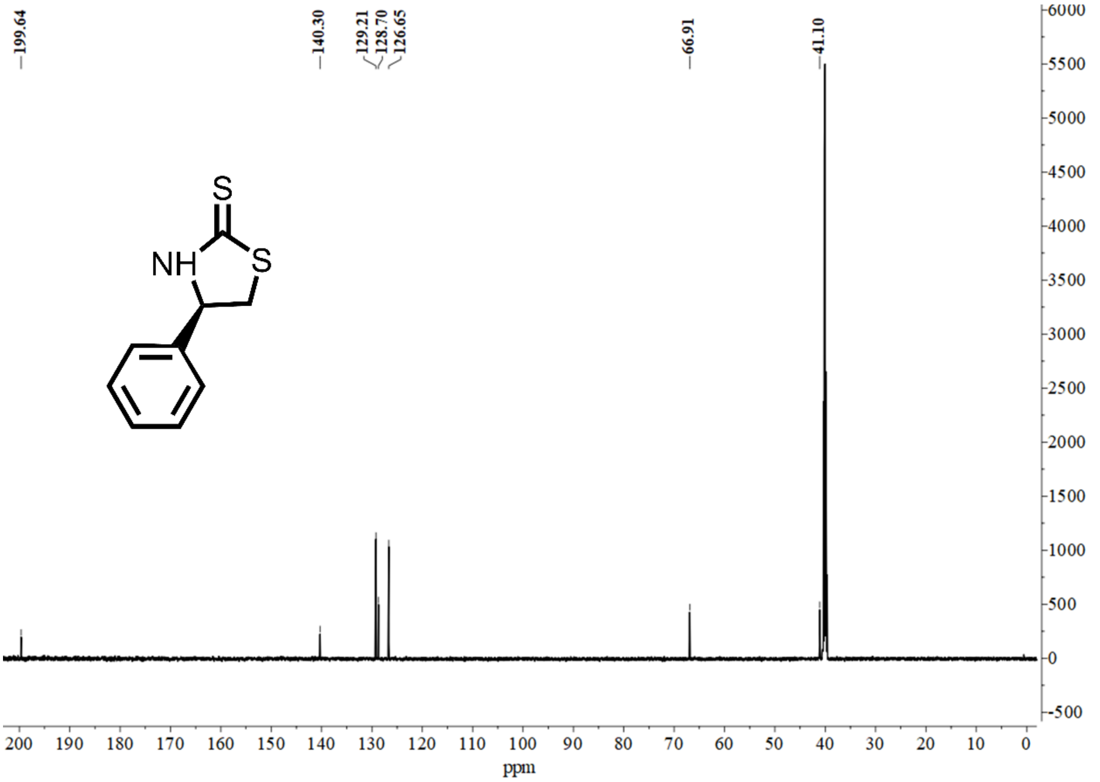

Figure S2. ^{13}C NMR spectrum of ^{R}Ph-H (151 MHz, DMSO-d_6).

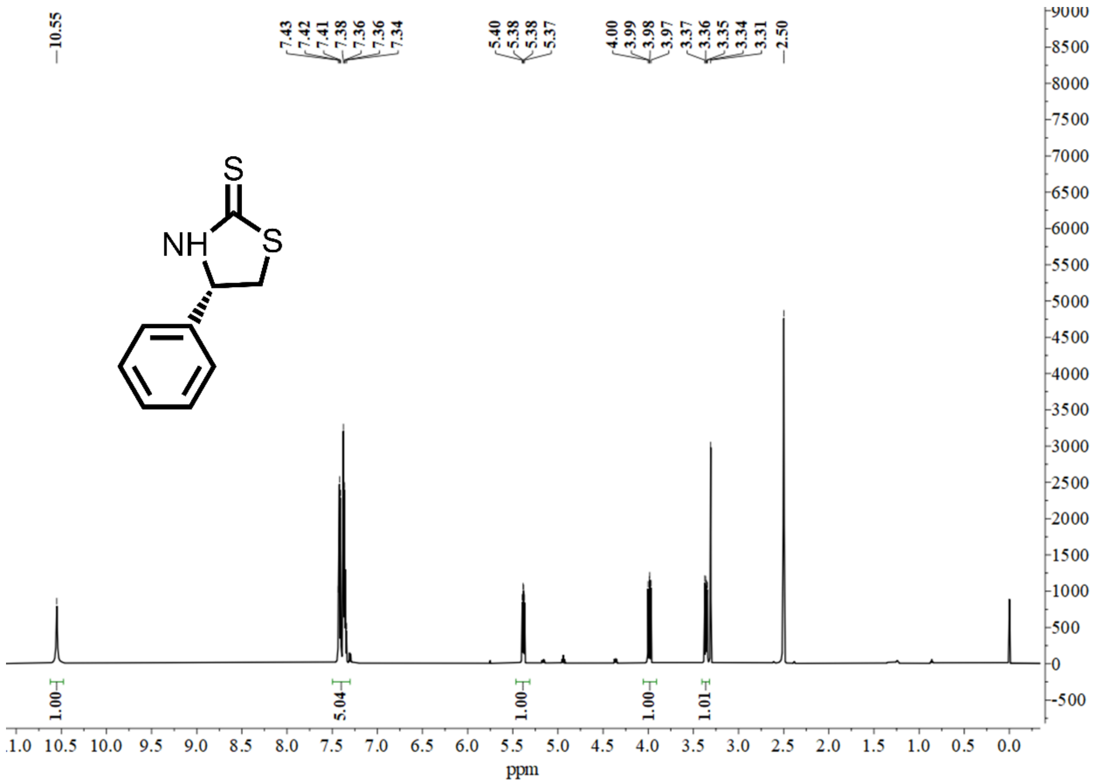

Figure S3. ^{1}H NMR spectrum of ^{S}Ph-H (600 MHz, DMSO-d_6).

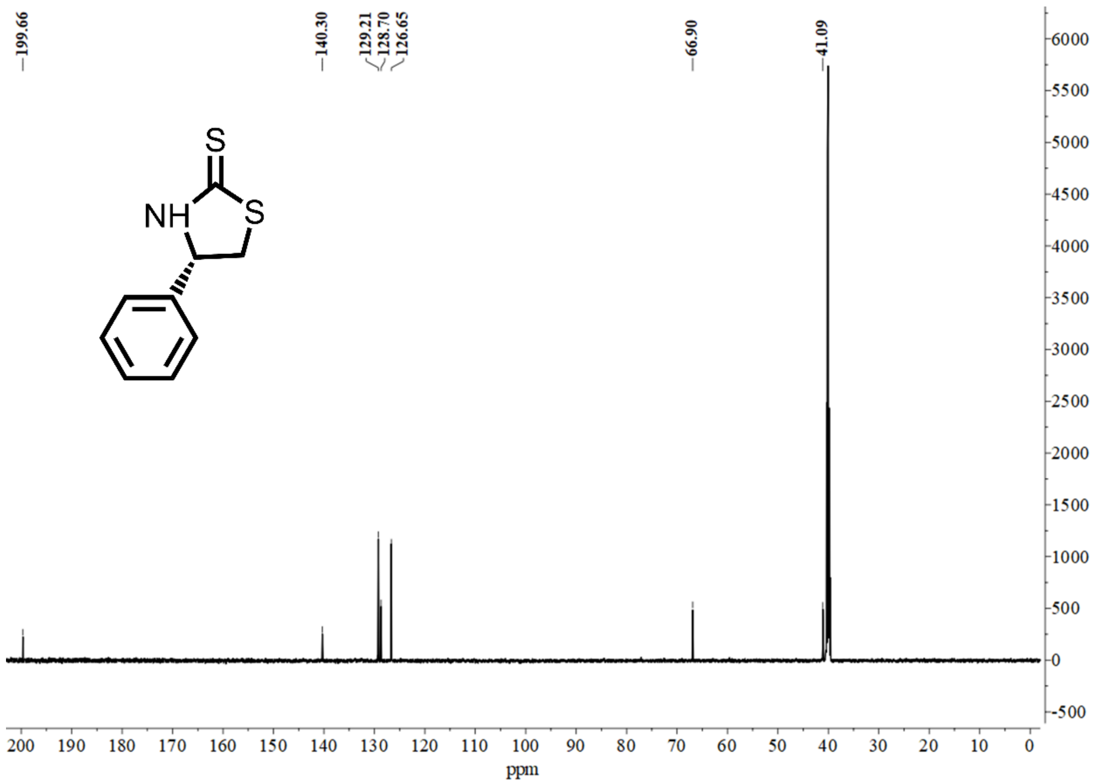

Figure S4. ^{13}C NMR spectrum of ^{S}Ph-H (151 MHz, DMSO-d_6).

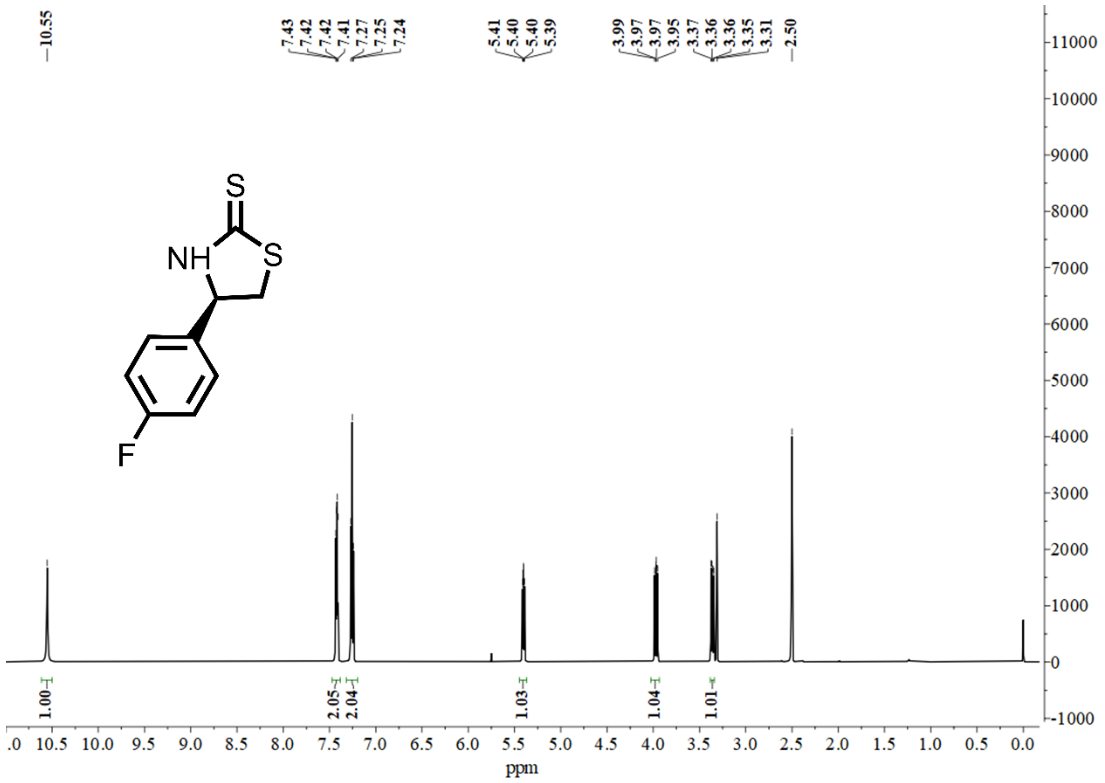

Figure S5. ^{1}H NMR spectrum of ^{R}Ph-F (600 MHz, DMSO-d_6).

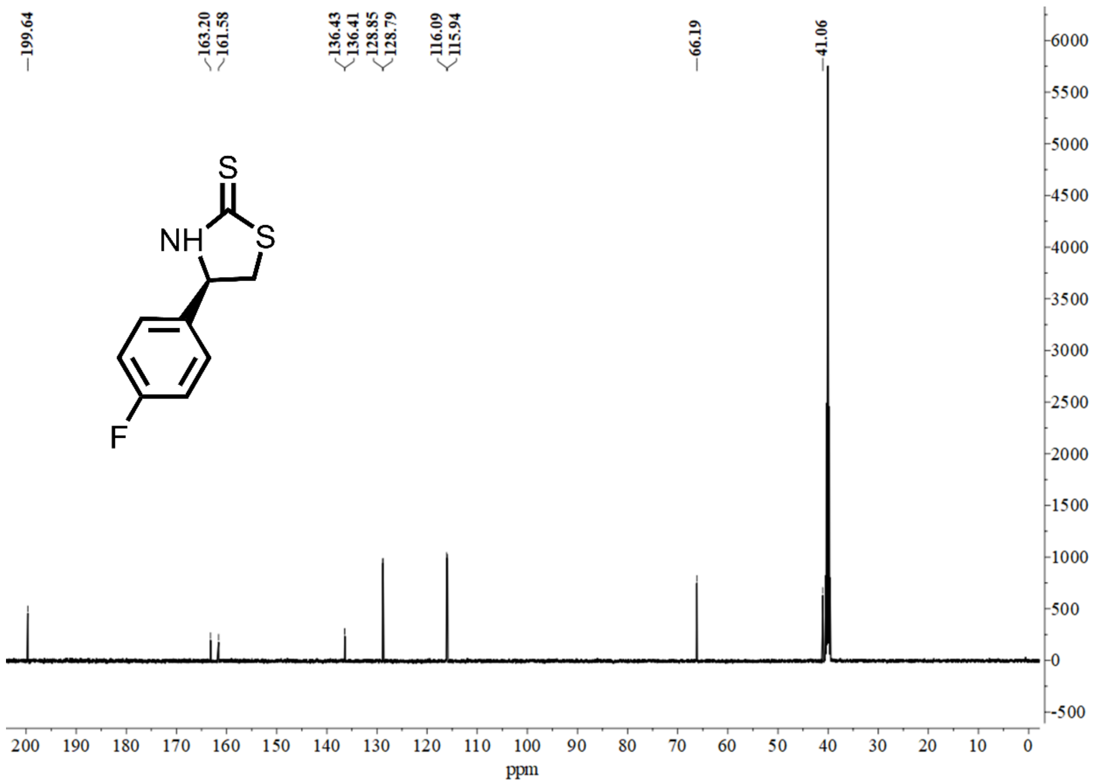

Figure S6. ^{13}C NMR spectrum of ^{R}Ph-F (151 MHz, DMSO-d_6).

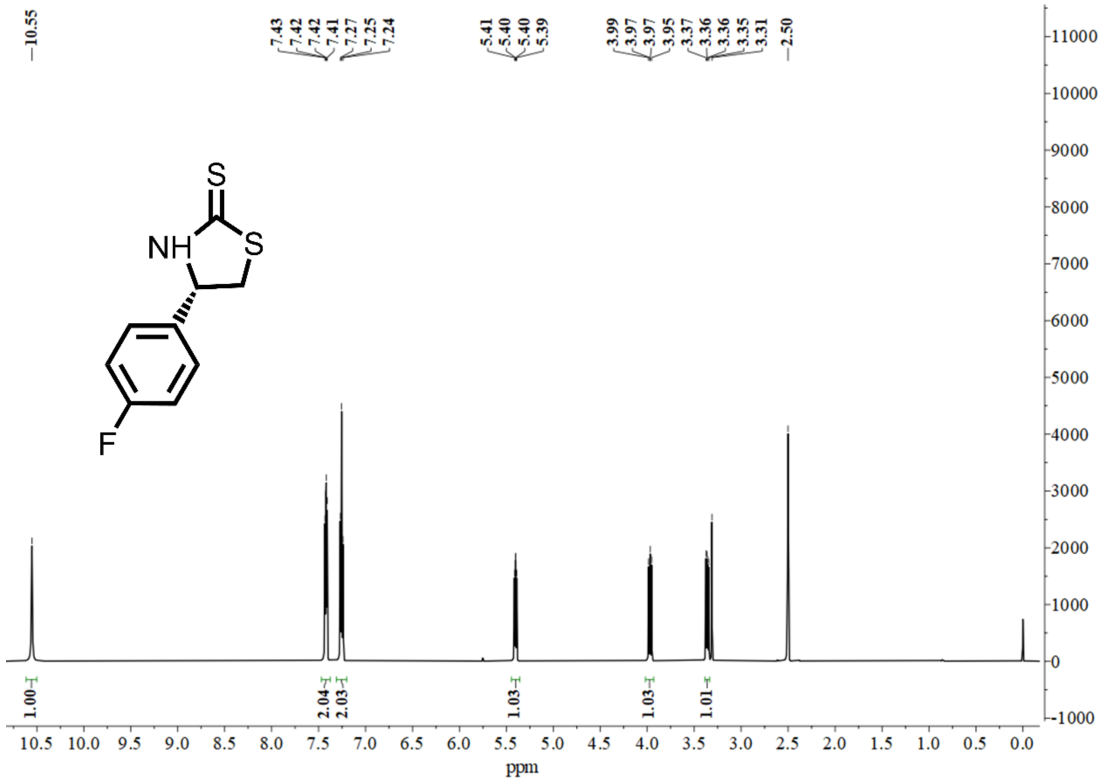

Figure S7. ^{1}H NMR spectrum of ^{S}Ph-F (600 MHz, DMSO-d_6).

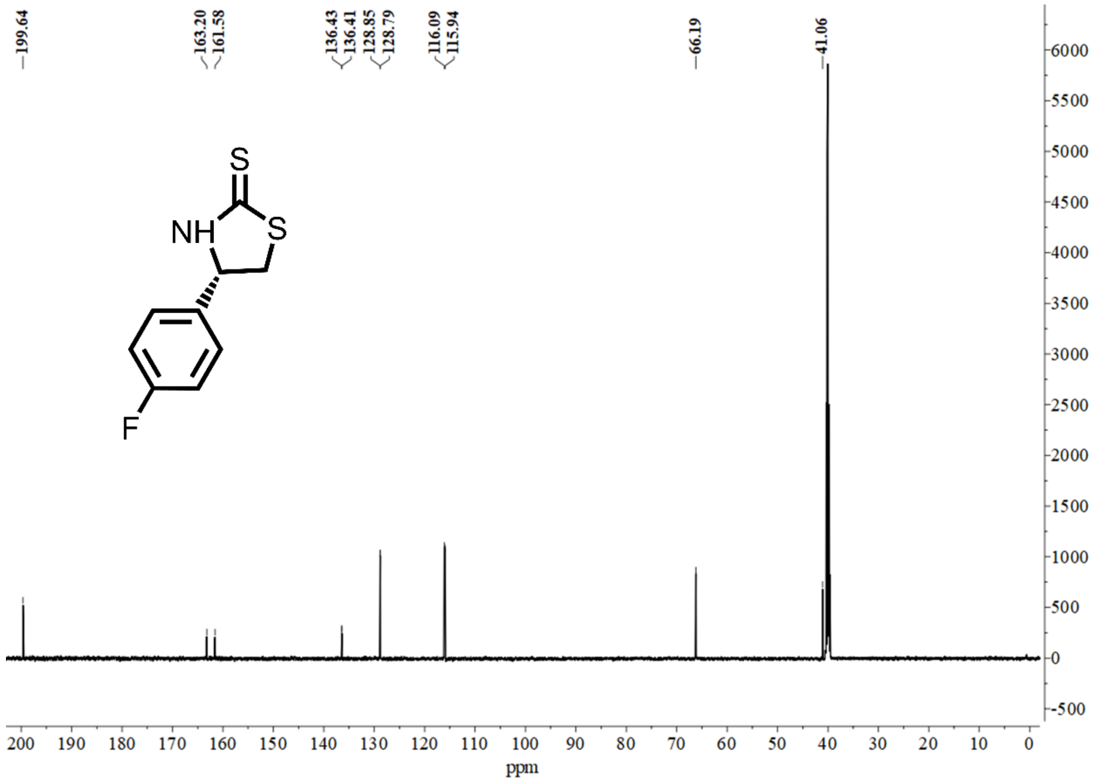

Figure S8. ^{13}C NMR spectrum of ^{S}Ph-F (151 MHz, DMSO-d_6).

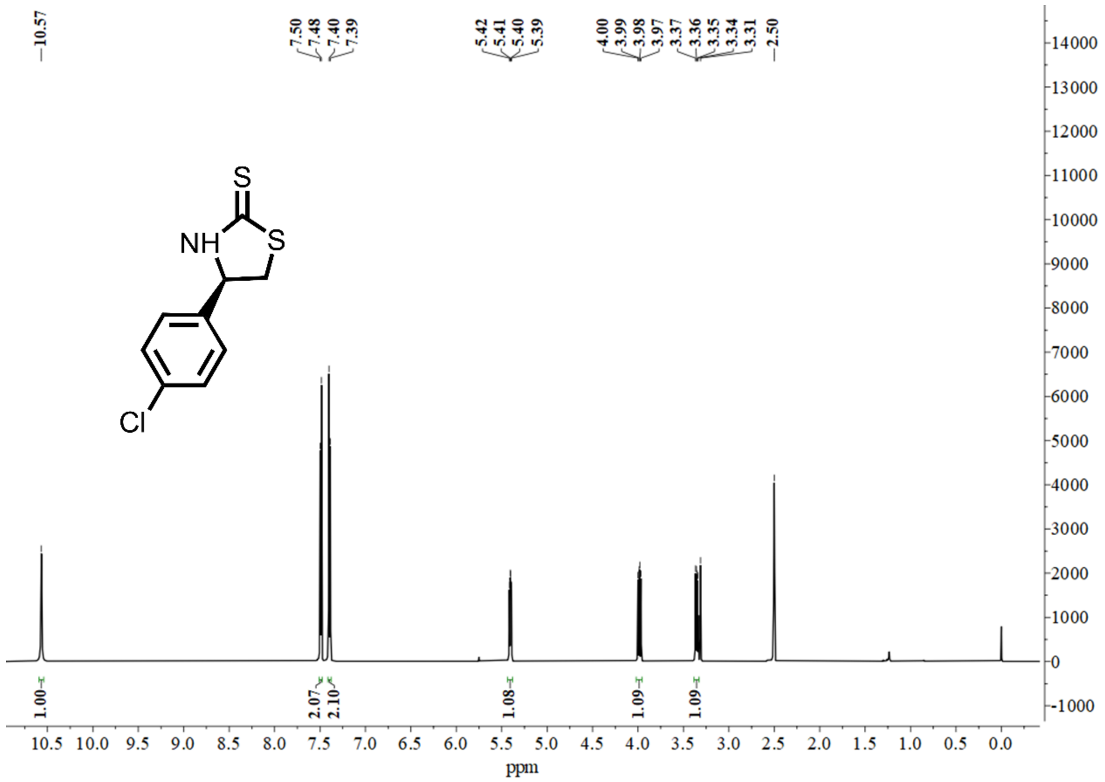

Figure S9. ^{1}H NMR spectrum of ^{R}Ph-Cl (600 MHz, DMSO-d_6).

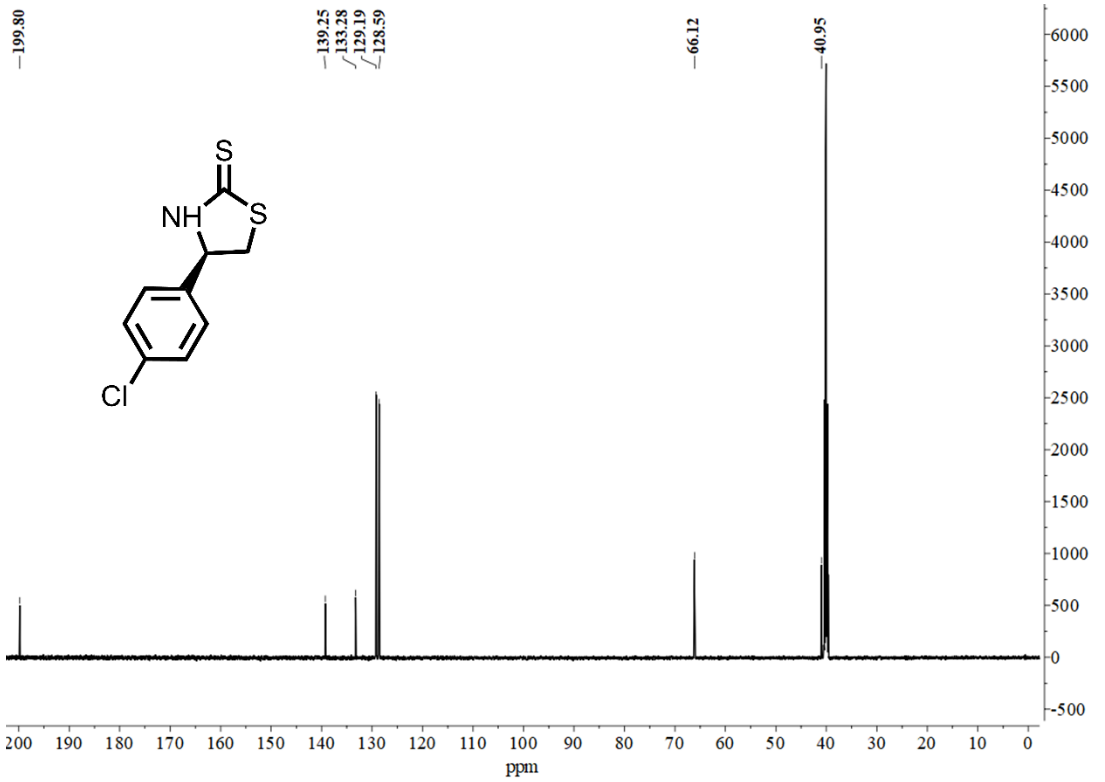

Figure S10. ^{13}C NMR spectrum of ^{R}Ph-Cl (151 MHz, DMSO-d_6).

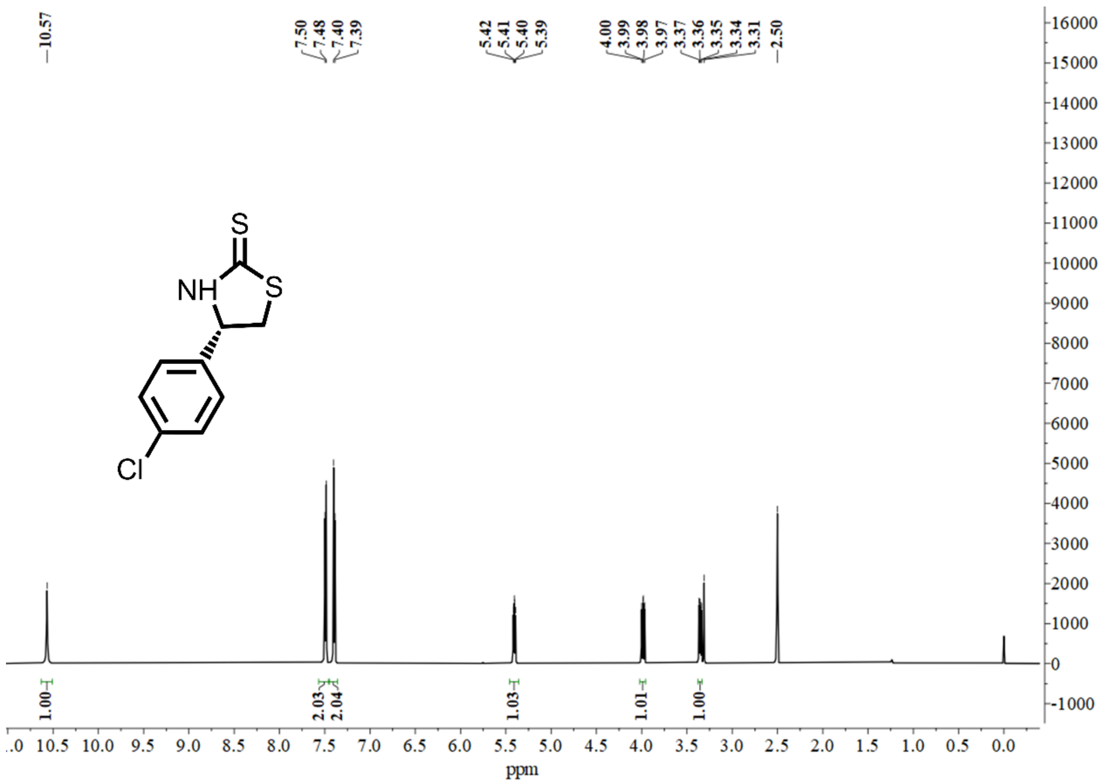

Figure S11. ^{1}H NMR spectrum of ^{S}Ph-Cl (600 MHz, DMSO-d_6).

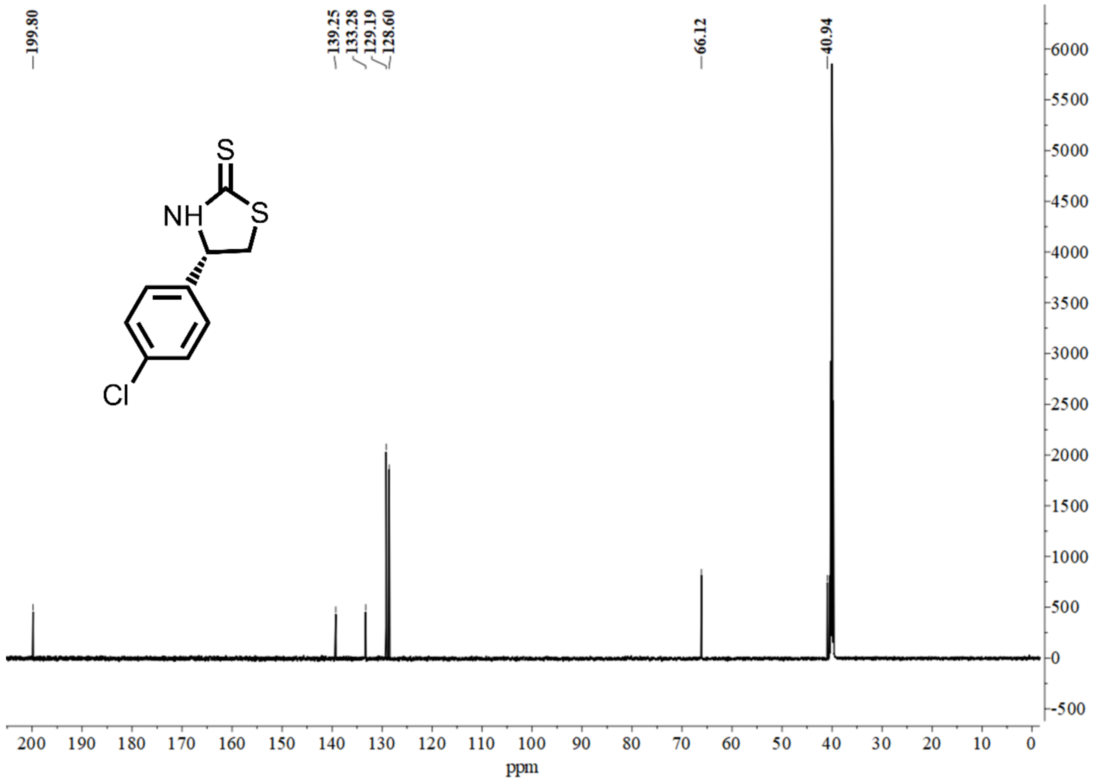

Figure S12. ^{13}C NMR spectrum of ^{S}Ph-Cl (151 MHz, DMSO-d_6).

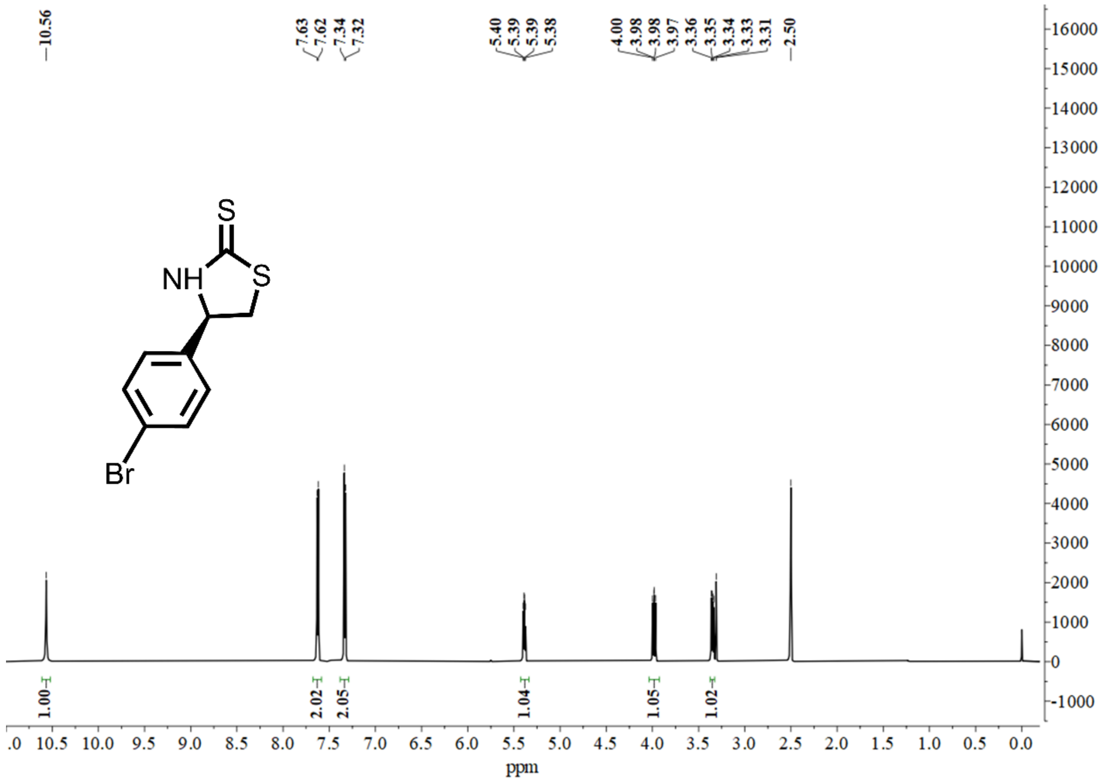

Figure S13. ^{1}H NMR spectrum of ^{R}Ph-Br (600 MHz, DMSO-d_6).

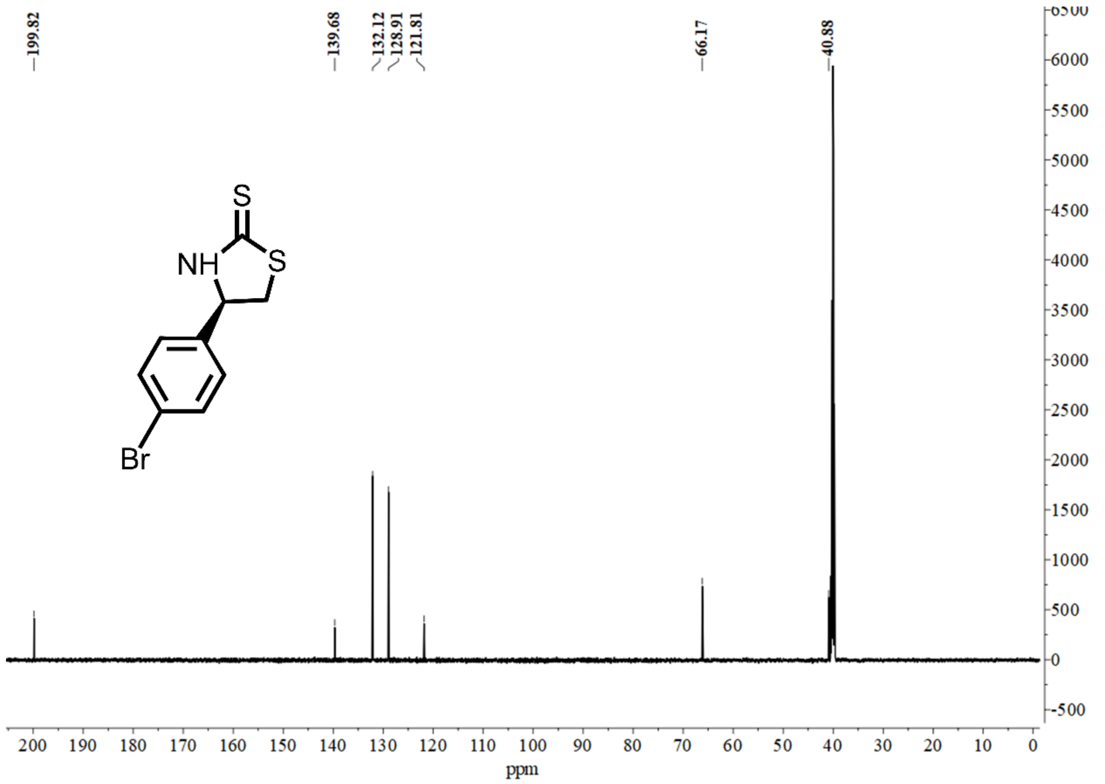

Figure S14. ^{13}C NMR spectrum of ^{R}Ph-Br (151 MHz, DMSO-d_6).

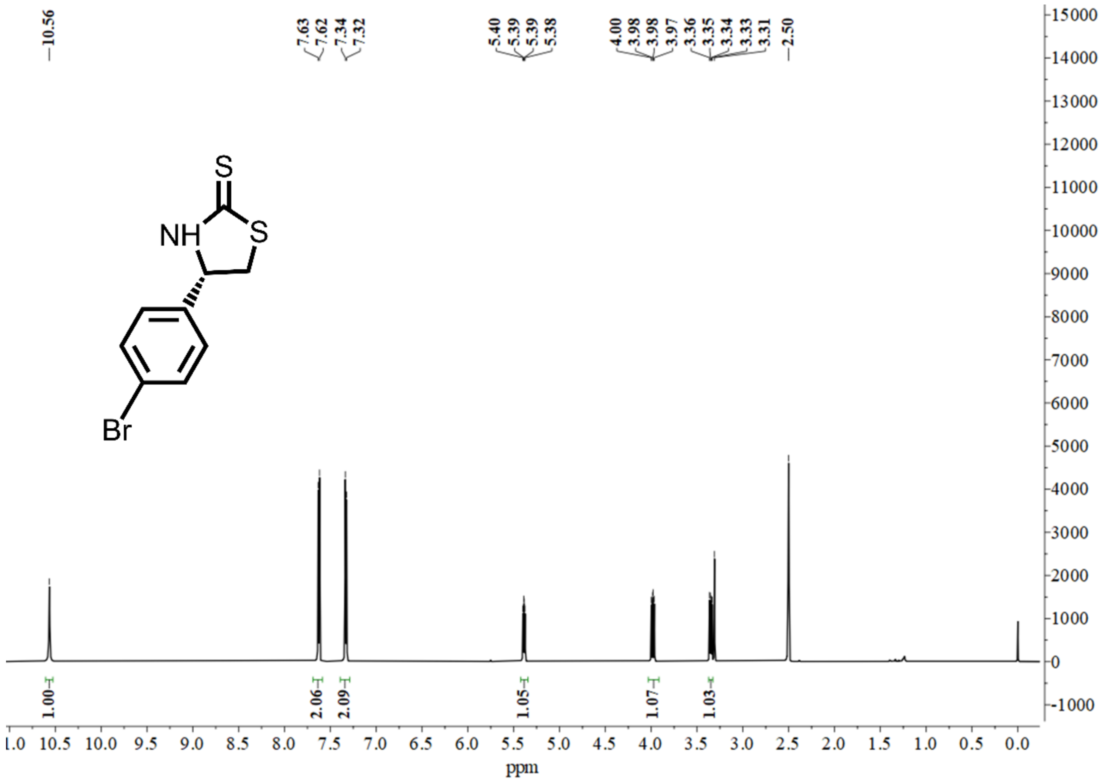

Figure S15. ^{1}H NMR spectrum of ^{S}Ph-Br (600 MHz, DMSO-d_6).

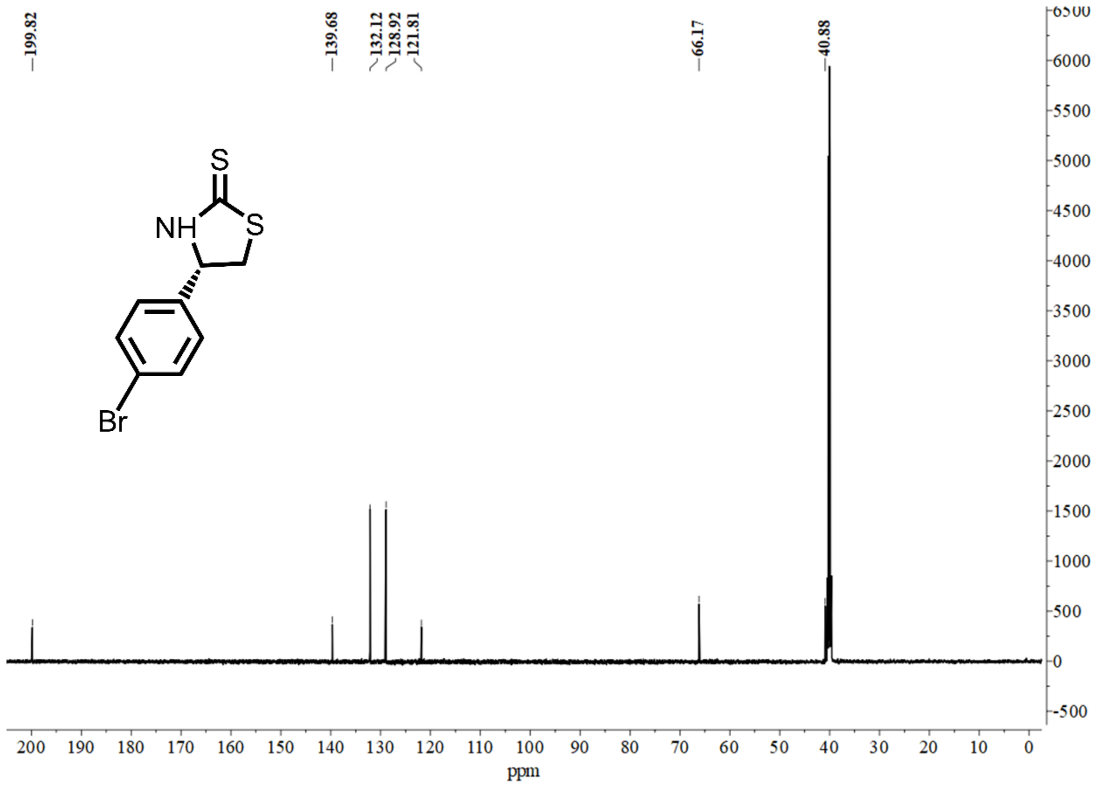

Figure S16. ^{13}C NMR spectrum of ^{S}Ph-Br (151MHz, DMSO-d_6).

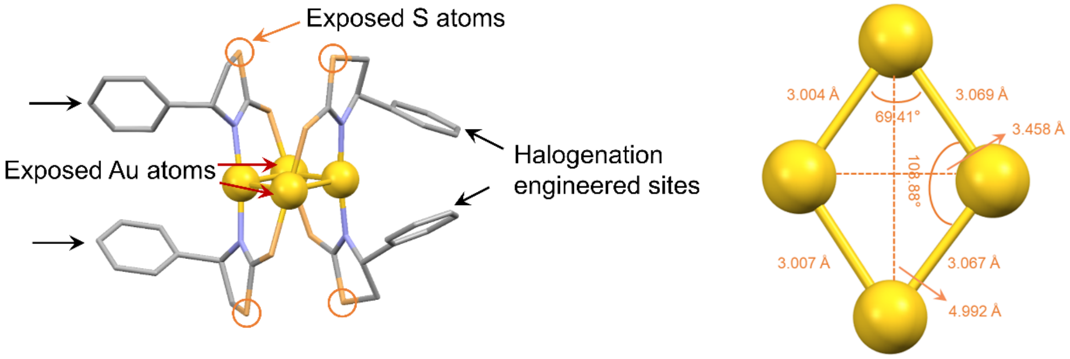

Figure S17. Analysis of the assembly site and rhombic metal core structure of the ${}^{R}Au_4$-H cluster (Color code: Au, golden yellow; C, dark gray; S, orange yellow; N, blue. Hydrogen atoms omitted for clarity).

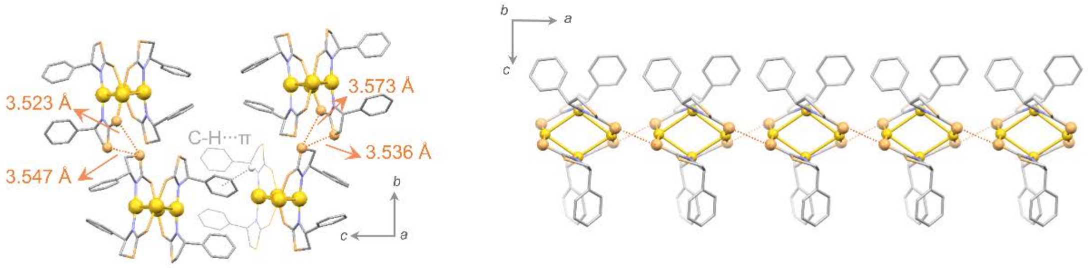

Figure S18. Additional S···S interactions between ${}^{R}Au_4$-H cluster molecules are shown (Color code: Au, golden yellow; C, dark gray; S, orange yellow; N, blue. Hydrogen atoms omitted for clarity).

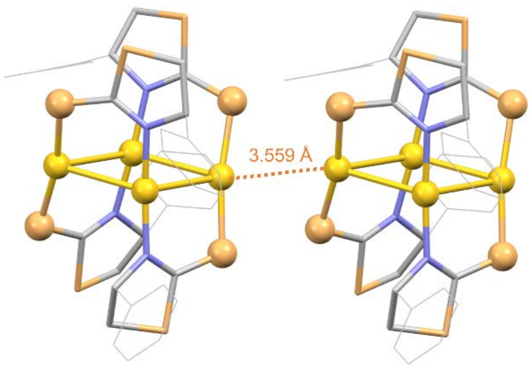

Figure S19. The Au···Au distance between adjacent ${}^{R}Au_4$-H clusters (Color code: Au, golden yellow; C, dark gray; S, orange yellow; N, blue. Hydrogen atoms omitted for clarity).

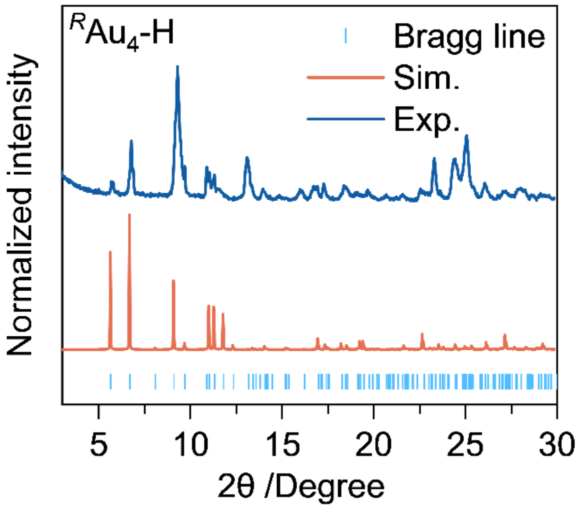

Figure S20. Powder XRD analysis of the ^{R}Au$_4$-H cluster.

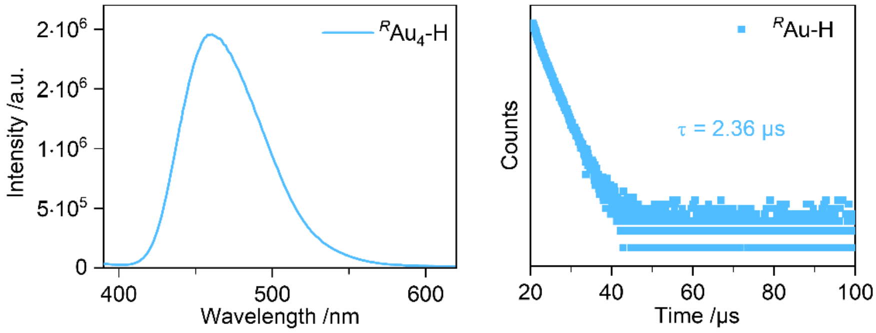

Figure S21. The emission spectrum and emission lifetime of ^{R}Au$_4$-H cluster crystals measured under 365 nm excitation.

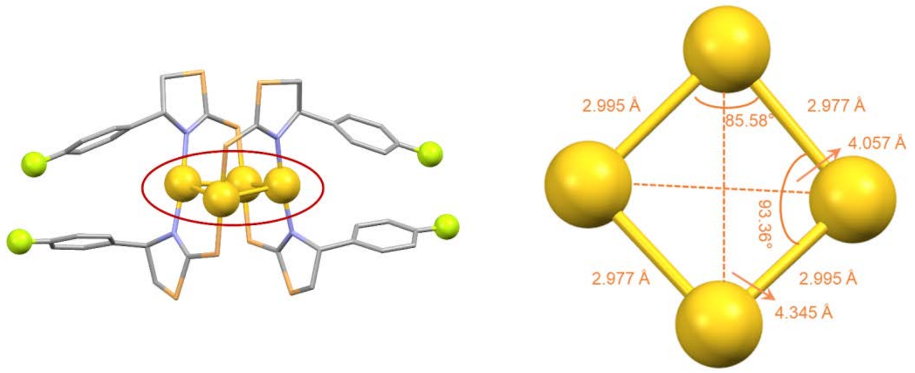

Figure S22. The single crystal structure of ^{R}Au$_4$-F and the analysis of its metal core (Color code: Au, golden yellow; C, dark gray; S, orange yellow; N, blue; F, light green. Hydrogen atoms omitted for clarity).

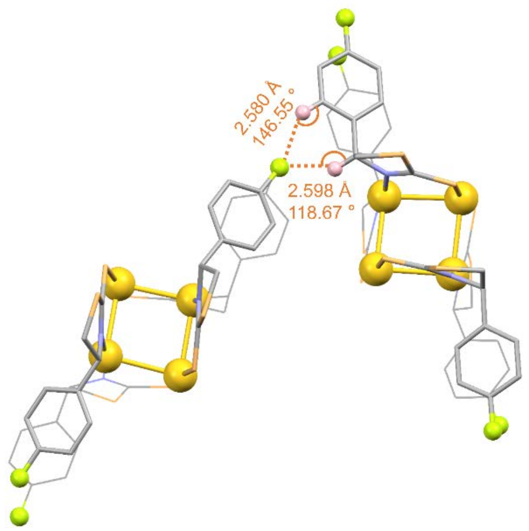

Figure S23. The C-H···F interactions between $^{R}Au_4$-F cluster molecules (Color code: Au, golden yellow; C, dark gray; S, orange yellow; N, blue; F, light green. Hydrogen atoms omitted for clarity).

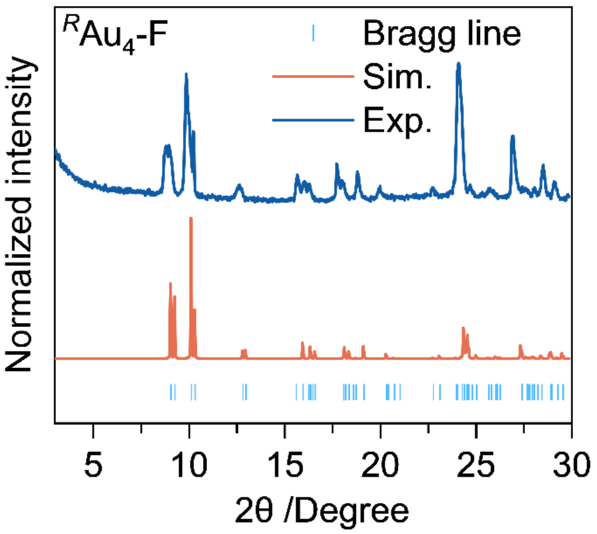

Figure S24. Powder XRD analysis of the $^{R}Au_4$-F cluster.

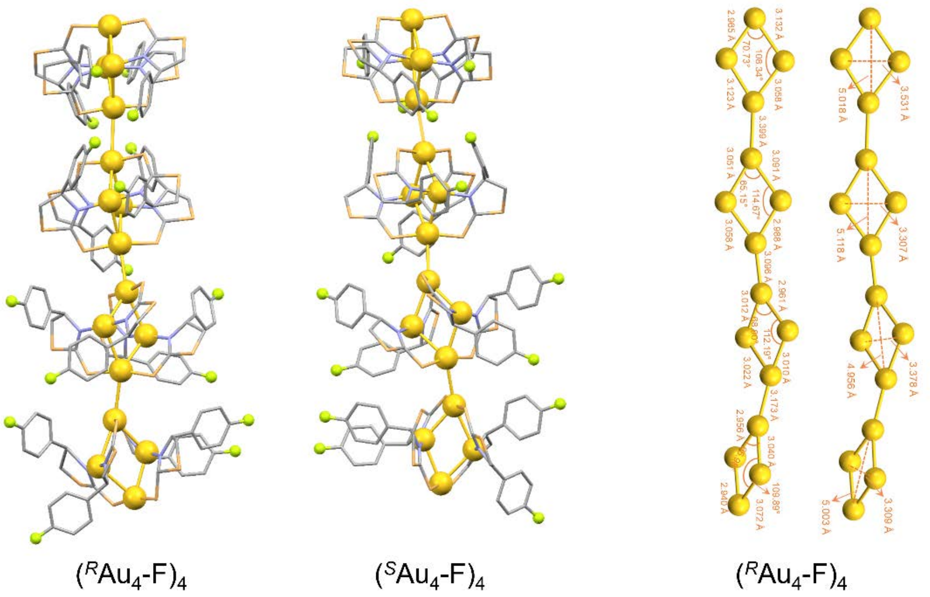

Figure S25. The complete crystal structure of (^{R}Au$_4$-F)$_4$ and (^{S}Au$_4$-F)$_4$ clusters (Color code: Au, golden yellow; C, dark gray; S, orange yellow; N, blue; F, light green. Hydrogen atoms omitted for clarity) and the analysis of the metal core of (^{R}Au$_4$-F)$_4$.

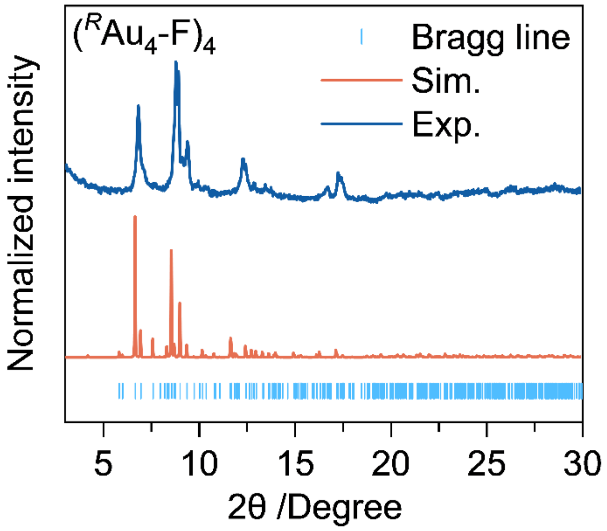

Figure S26. Powder XRD analysis of the (^{R}Au$_4$-F)$_4$ cluster.

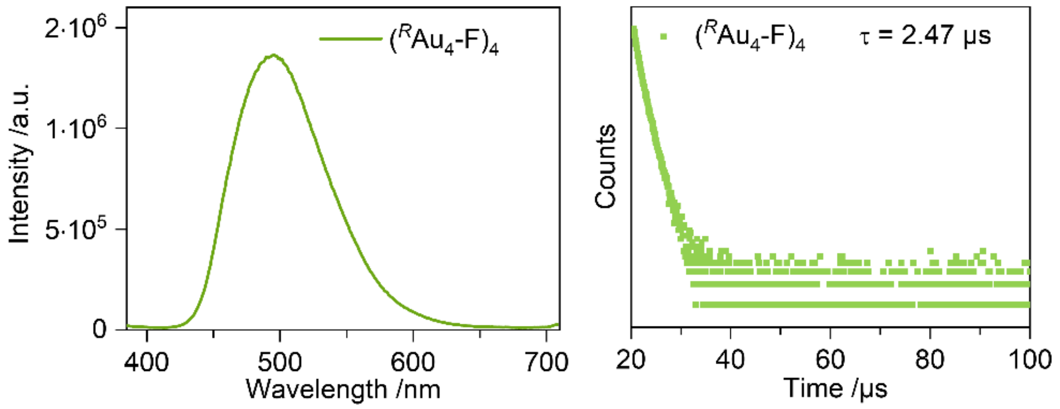

Figure S27 The emission spectrum and emission lifetime of $(^{R}Au_4\text{-}F)_4$ cluster crystals measured under 365 nm excitation.

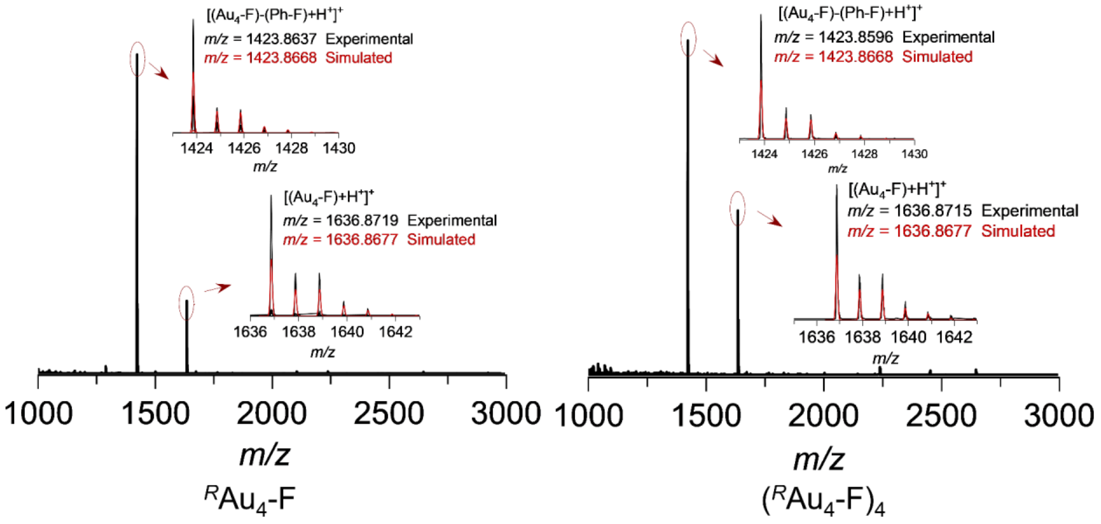

Figure S28. Positive mode ESI-MS of $^{R}Au_4\text{-}F$ and $(^{R}Au_4\text{-}F)_4$ dissolved in DMF/CH_3CN. Insets: Enlarged portion of the ESI-MS exhibiting the measured (black line) and simulated (red line) isotopic distribution patterns in the *m*/*z* range of 1000-3000 with a charge state of +1.

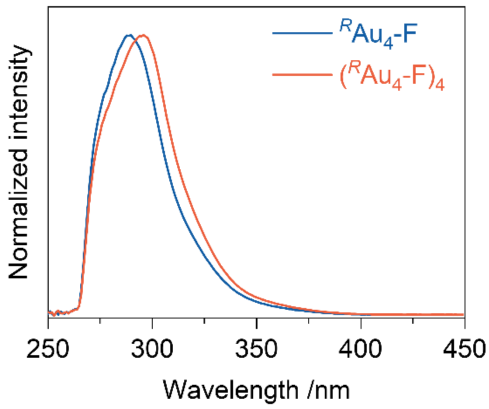

Figure S29. UV-Vis spectra of ${}^{R}Au_4$-F (1.75×10^{-4} mol/L) and (${}^{R}Au_4$-F)$_4$ (4.4×10^{-5} mol/L) in DMF.

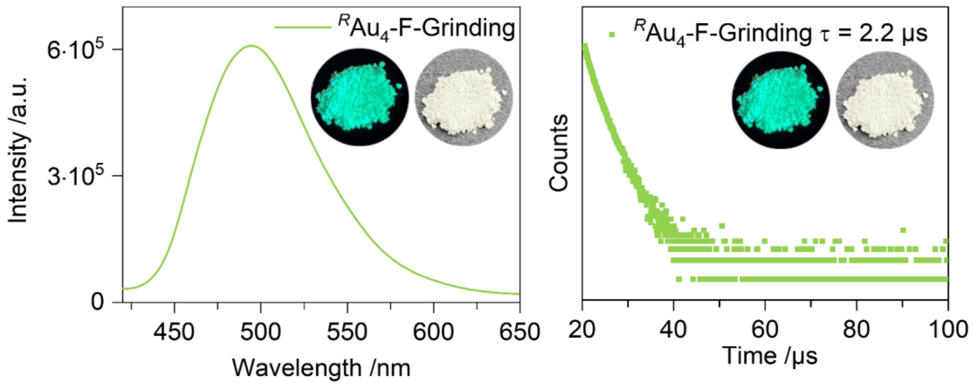

Figure S30. The emission spectrum and emission lifetime of ${}^{R}Au_4$-F-Grinding measured under 365 nm excitation.

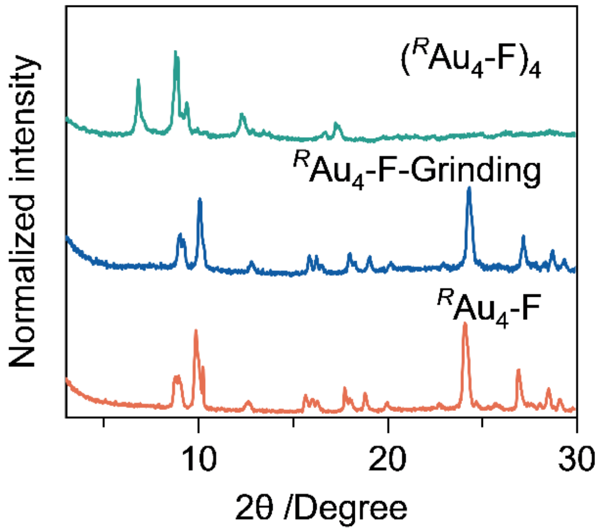

Figure S31. Comparative XRD analysis ${}^{R}Au_4$-F, ${}^{R}Au_4$-F-Grinding and (${}^{R}Au_4$-F)$_4$.

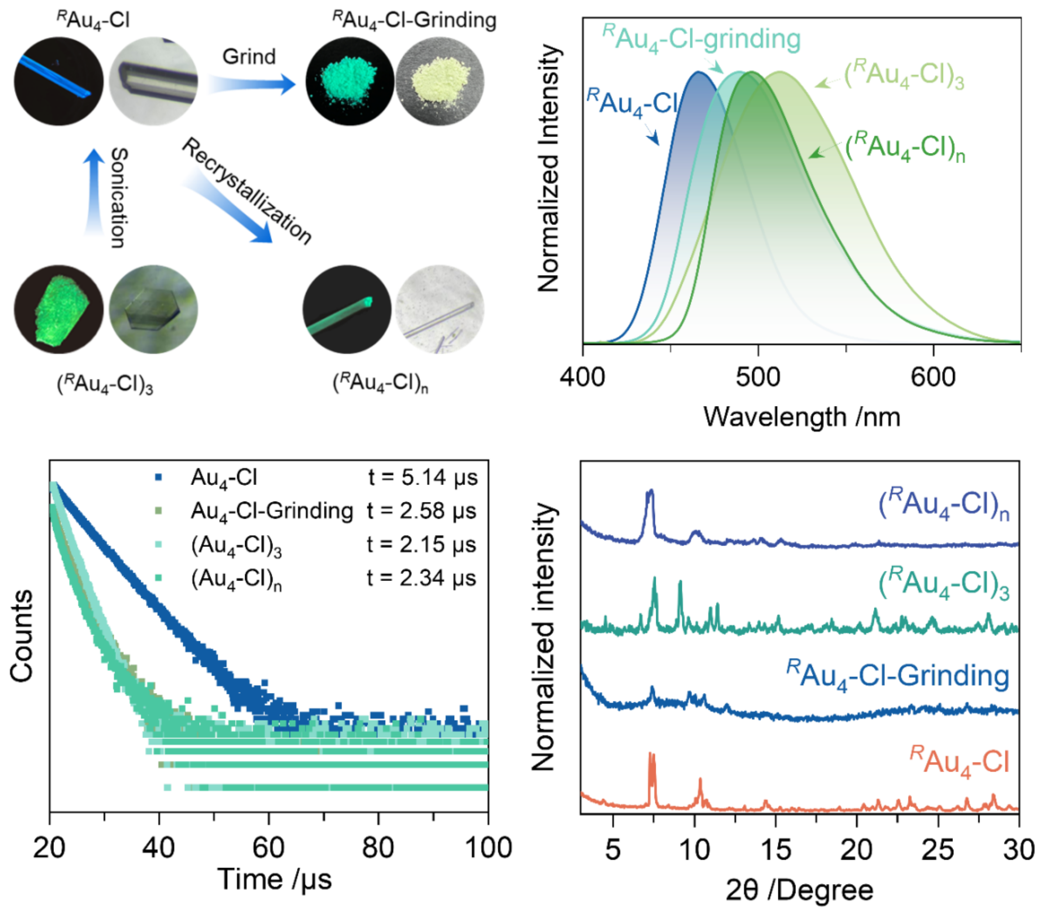

Figure S32. The structural transformation pathways among ${}^{R}Au_4$-Cl, ${}^{R}Au_4$-Cl-Grinding, $({}^{R}Au_4\text{-Cl})_3$, and $({}^{R}Au_4\text{-Cl})_n$ are elucidated (under 365 nm UV and visible light), along with a systematic comparison of their emission spectrum (under 365 nm excitation), emission lifetimes and XRD spectra.

To further investigate the generality of the luminescence redshift induced by mechanical grinding in the ${}^{R}Au_4$-X (X = F, Cl, Br) cluster system[6]. The blue-emitting ${}^{R}Au_4$-Cl cluster was subjected to mechanical grinding, and the resulting sample was designated as ${}^{R}Au_4$-Cl-Grinding. Experimental results demonstrate that prolonged grinding not only transforms the sample color from colorless and transparent to yellow but also induces emission redshift of approximately 25 nm, resulting in green luminescence with a photoluminescence quantum yield of 20.28%. As illustrated in the Supplementary Fig. 32, comparative analysis of the emission spectra and lifetimes of ${}^{R}Au_4$-Cl, ${}^{R}Au_4$-Cl-Grinding, $({}^{R}Au_4\text{-Cl})_3$, and $({}^{R}Au_4\text{-Cl})_n$ clearly reveals distinct photophysical differences among these species. To elucidate

the structural origin of the luminescence modulation, a systematic comparison was conducted on the XRD patterns of four samples. As illustrated in the Supplementary Fig. 32, The main diffraction peak of $^{R}Au_4$-Cl crystals evolved from a doublet to a singlet upon mechanical grinding, indicating partial disruption of its crystalline order upon mechanical grinding [7,8]. While the positions of the remaining diffraction peaks remained largely consistent with those of pristine $^{R}Au_4$-Cl, they exhibited a slight shift toward lower 2θ angles, contrasting sharply with the higher 2θ shifts (indicative of lattice contraction) observed in $^{R}Au_4$-F-Grinding. This discrepancy can be attributed to the nature of the halogen, Cl^- possesses a larger ionic radius and enhanced coordination flexibility, which facilitate anisotropic lattice expansion under mechanical stress. Such structural distortion induces elongation of Au–Au bonds, thereby weakening metal-centered electronic coupling, reducing the d-d transition energy, and ultimately resulting in a 25 nm emission redshift[9,10]. Furthermore, crystal growth kinetics studies reveal that $(^{R}Au_4\text{-}Cl)_3$ acts as a metastable assembly intermediate[11], which rapidly converts into the thermodynamically stable $^{R}Au_4$-Cl phase upon ultrasonic treatment of the mother liquor, underscoring its high solution-phase reactivity and intrinsic structural lability. These insights establish a critical foundation for precise control over crystal nucleation and growth, as well as rational optimization of synthetic protocols. Leveraging this structural and mechanistic understanding, high-quality $(^{R}Au_4\text{-}Cl)_n$ single crystals were successfully grown via solvent displacement recrystallization. The resultant crystals exhibit excellent crystallinity, enabling accurate characterization of molecular packing motifs, interlayer stacking arrangements, and excited-state properties, thus establishing a robust materials platform for future applications in optoelectronic devices and chiral optical sensing technologies.

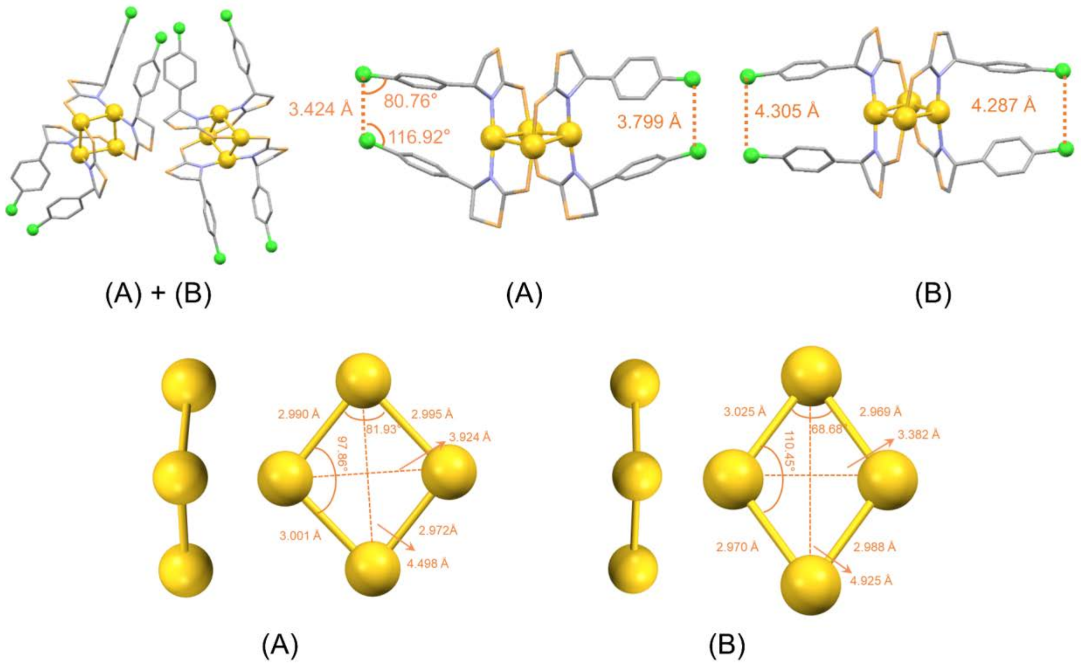

Figure S33. Crystal structure of two ${}^{R}Au_4$-Cl molecules (clusters A and B) in the asymmetric unit and analysis of their metal core (Color code: Au, golden yellow; C, dark gray; S, orange yellow; N, blue; Cl, green. Hydrogen atoms omitted for clarity).

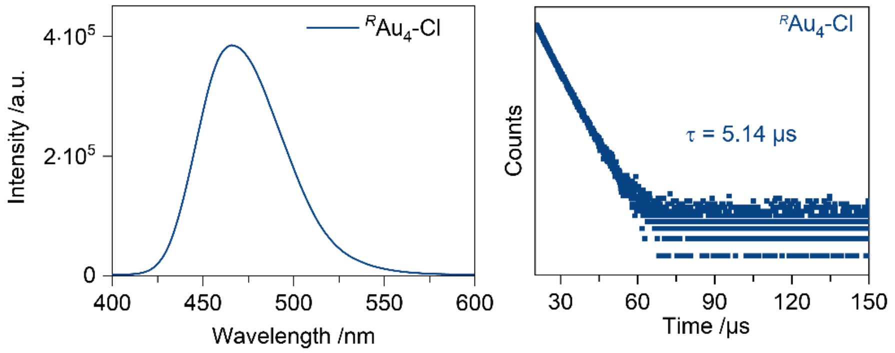

Figure S34. The emission spectrum and emission lifetime of ${}^{R}Au_4$-Cl cluster crystals measured under 365 nm excitation.

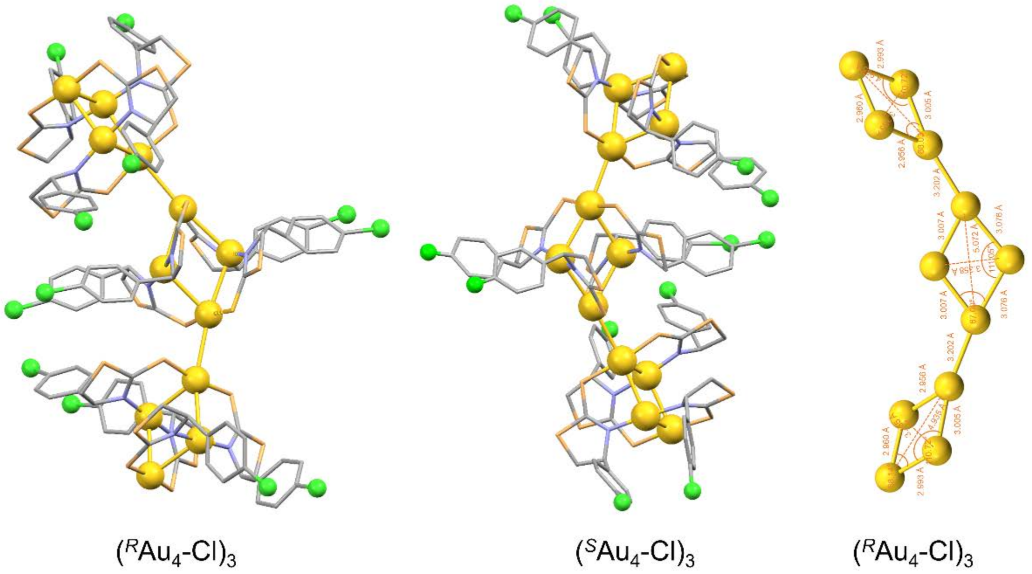

Figure S35. The complete crystal structure of (^{R}Au$_4$-Cl)$_3$ and (^{S}Au$_4$-Cl)$_3$ clusters and the analysis of the metal core of (^{R}Au$_4$-Cl)$_3$. (Color code: Au, golden yellow; C, dark gray; S, orange yellow; N, blue; Cl, green. Hydrogen atoms omitted for clarity).

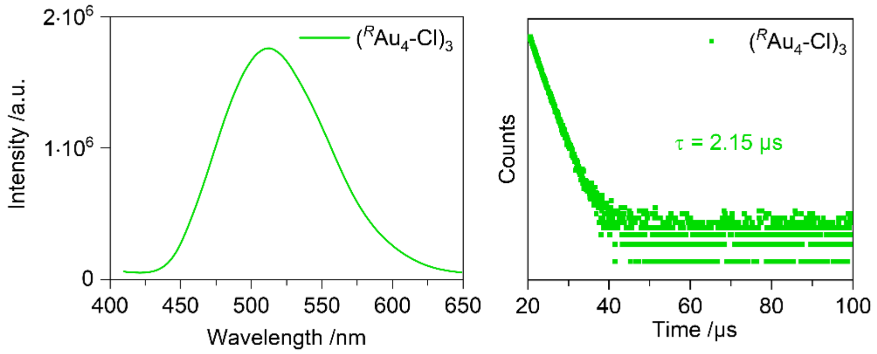

Figure S36. The emission spectrum and emission lifetime of (^{R}Au$_4$-Cl)$_3$ cluster crystals measured under 365 nm excitation.

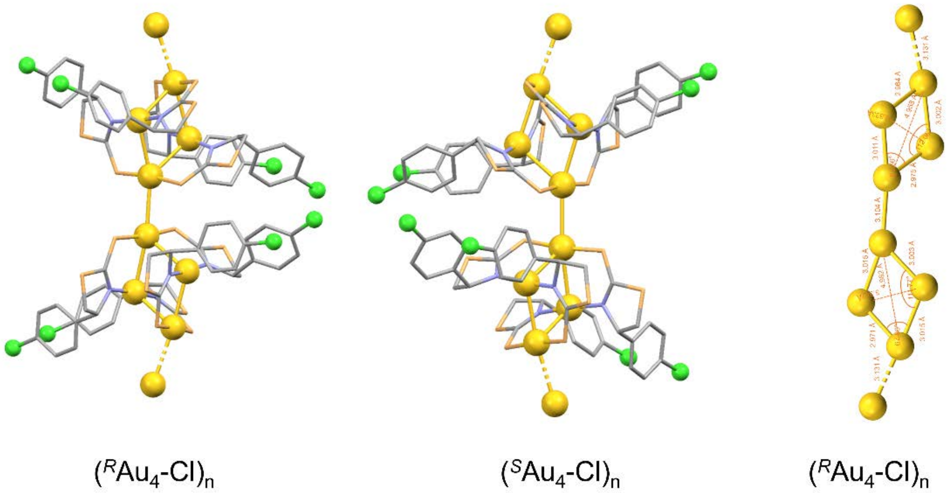

Figure S37. The complete crystal structure of (^{R}Au$_4$-Cl)$_n$ and (^{S}Au$_4$-Cl)$_n$ clusters and the analysis of the metal core of (^{R}Au$_4$-Cl)$_n$ (Color code: Au, golden yellow; C, dark gray; S, orange yellow; N, blue; Cl, green. Hydrogen atoms omitted for clarity).

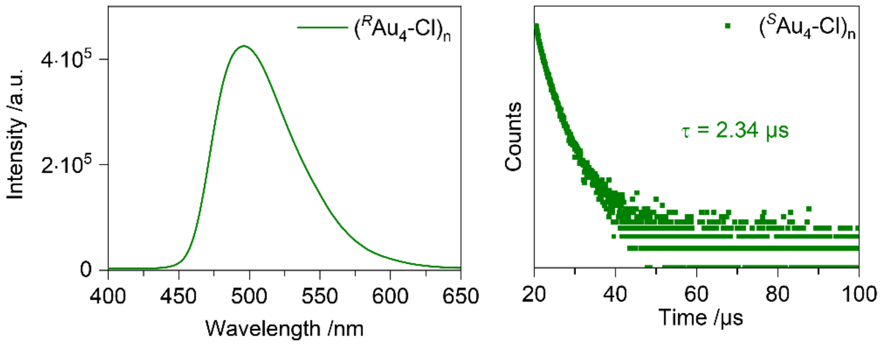

Figure S38. The emission spectrum and emission lifetime of (^{R}Au$_4$-Cl)$_n$ cluster crystals measured under 365 nm excitation.

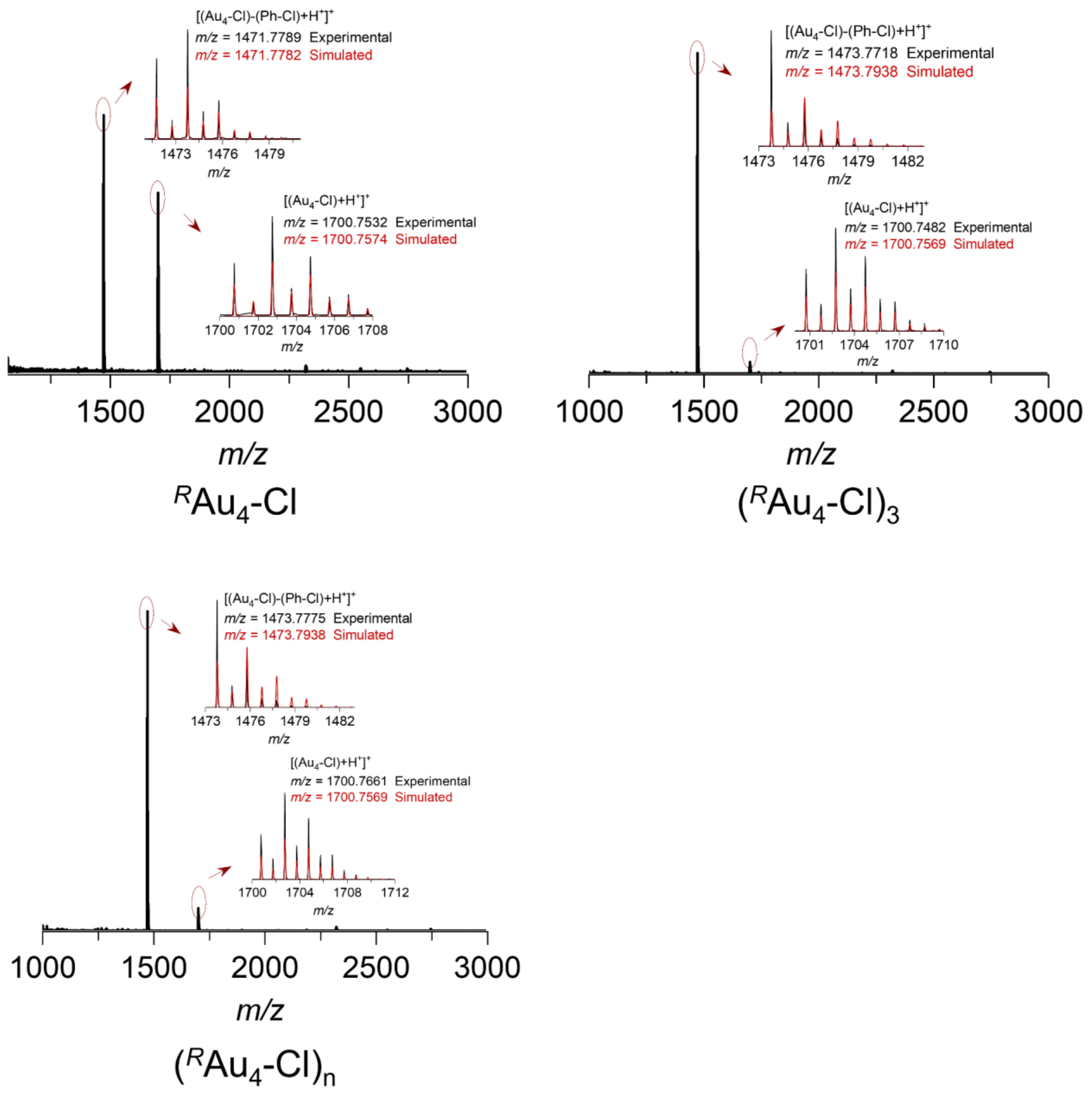

Figure S39. Positive mode ESI-MS of ^{R}Au$_4$-Cl, (^{R}Au$_4$-Cl)$_3$ and (^{R}Au$_4$-Cl)$_n$ dissolved in DMF/CH$_3$CN. Insets: Enlarged portion of the ESI-MS exhibiting the measured (black line) and simulated (red line) isotopic distribution patterns in the *m*/*z* range of 1000-3000 with a charge state of +1.

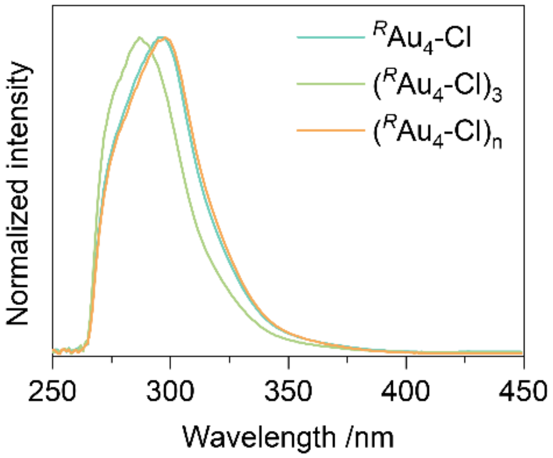

Figure S40. UV-Vis spectra of ^{R}Au$_4$-Cl (1.7×10^{-4} mol/L), (^{R}Au$_4$-Cl)$_3$ (5.7×10^{-5} mol/L) and (^{R}Au$_4$-Cl)$_n$ (8.5×10^{-5} mol/L) in DMF.

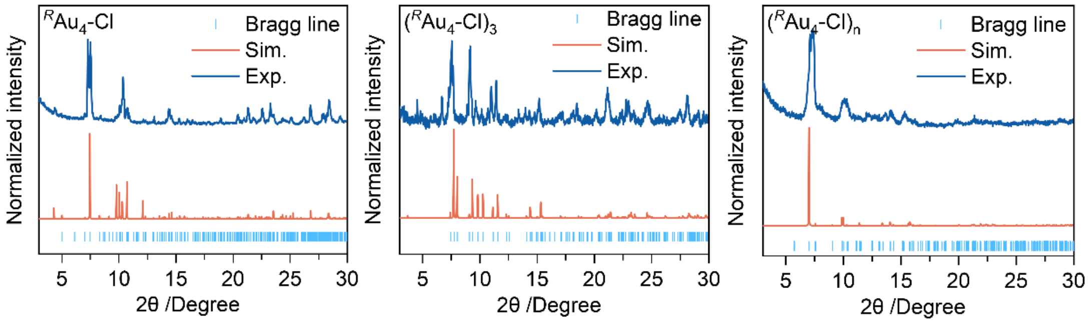

Figure S41. Comparative XRD analysis ^{R}Au$_4$-Cl, (^{R}Au$_4$-Cl)$_3$ and (^{R}Au$_4$-Cl)$_n$.

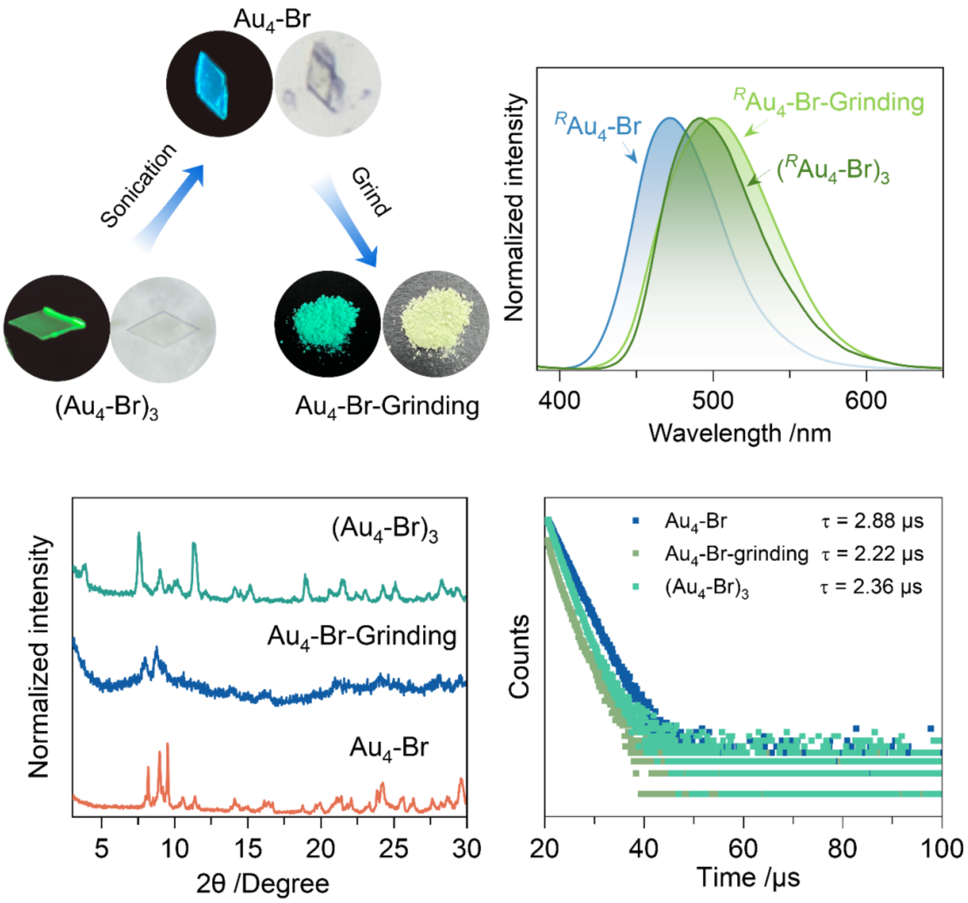

Figure S42. The structural transformation pathways among $^{R}Au_4$-Br, $^{R}Au_4$-Br-Grinding and ($^{R}Au_4$-Br)$_3$ were systematically elucidated (under 365 nm UV and visible light), along with a comparative analysis of their emission spectra and emission lifetimes (under 365 nm excitation) and XRD spectra.

Similarly, the $^{R}Au_4$-Br cluster exhibits a comparable mechanochromic luminescence[12] behavior. Specifically, prolonged mechanical grinding not only transforms the sample color from colorless and transparent to yellow but also induces a redshift in the emission wavelength, shifting the fluorescence from blue to green, with a photoluminescence quantum yield of 14.66%, the ground sample is designated as $^{R}Au_4$-Br-Grinding. As illustrated in the Supplementary Fig. 42, comparative analysis of the emission spectra and luminescence lifetimes of $^{R}Au_4$-Br, $^{R}Au_4$-Br-Grinding, and ($^{R}Au_4$-Br)$_3$ clearly reveals distinct photophysical differences among these species. XRD results reveal that the main diffraction peak of $^{R}Au_4$-Br-Grinding has undergone noticeable broadening and attenuation, which

indicates partial disruption of the long-range crystalline order caused by mechanical grinding. Meanwhile, the remaining diffraction peaks collectively shift toward lower 2θ angles. This feature is consistently observed in $^{R}Au_4$-Cl-Grinding and further underscores the critical influence of halogen identity on lattice response under mechanical stimulation. This peak shift likely originates from mechanical force-induced lattice expansion, which perturbs the electronic structure of the clusters and consequently results in the observed emission redshift. Furthermore, green-emitting $^{R}Au_4$-Br crystals grown from a dichloromethane/acetone mixed solvent system spontaneously convert into blue-emitting $^{R}Au_4$-Br monomers upon prolonged ultrasonic treatment of the mother liquor. This transformation demonstrates that $(^{R}Au_4\text{-Br})_3$ is thermodynamically unstable in solution and preferentially evolves into the more stable monomeric form. These clusters exhibit outstanding optical and chiroptical properties, holding great promise for applications in optoelectronic functional materials and emerging chiral catalytic systems.

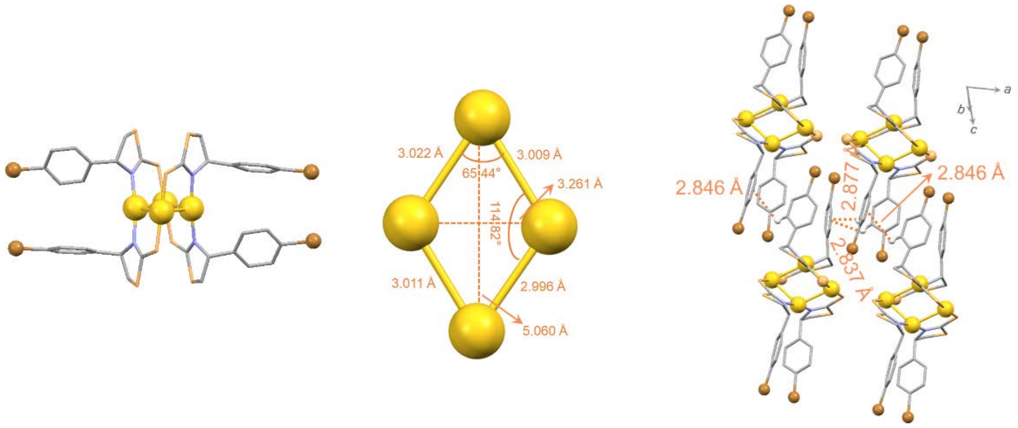

Figure S43. The crystal structure, core structural analysis, and intermolecular C-H···π interactions of ^{R}Au$_4$-Br (Color code: Au, golden yellow; C, dark gray; S, orange yellow; N, blue; Br, tawny. Hydrogen atoms omitted for clarity).

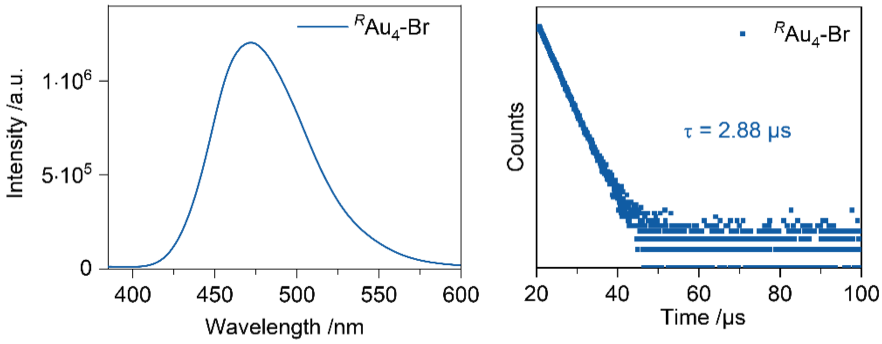

Figure S44. The emission spectrum and emission lifetime of ^{R}Au$_4$-Br cluster crystals measured under 365 nm excitation.

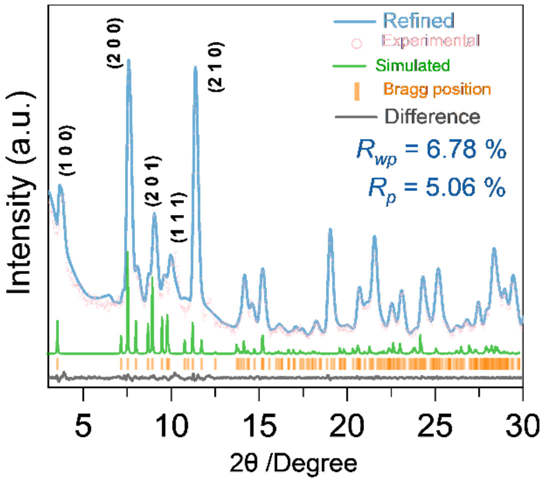

Figure S45. Experimental (red dot) and simulated (blue line) PXRD patterns of (^{R}Au$_4$-Br)$_3$.

To investigate the crystalline structure of (^{R}Au$_4$-Br)$_3$, Powder X-ray Diffraction (PXRD) analysis was performed alongside theoretical structural simulations using Materials Studio. According to the theoretical simulations and Pawley refinement, (^{R}Au$_4$-Br)$_3$ and (^{R}Au$_4$-Cl)$_3$ are isomorphic models with the following unit-cell parameters: a = 23.5802 Å, b = 10.8164 Å, c = 24.4851 Å, $\alpha = \gamma$ = 90.00°, β = 95.75° and the structure was classified under the $C2$ space group. The residual factors, R_p = 5.06% and R_{wp} = 6.78%, validate the accuracy of the computational model (Supplementary Fig. 45). The PXRD pattern of (^{R}Au$_4$-Br)$_3$ reveals characteristic peaks at 3.63°, 7.53°, 9.11°, 9.84°and 11.41°, which correspond to the (0, 0, 1), (2, 0, 0), (1, 0, 0), (1, 1, 1), and (1, 1, -2) crystal planes, respectively.

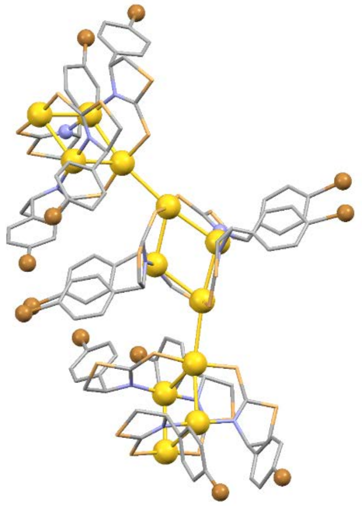

($(^{R}Au_4\text{-}Br)_3$)

Figure S46. The complete crystal structure of the $(^{R}Au_4\text{-}Br)_3$ cluster derived from structural simulations (Color code: Au, golden yellow; C, dark gray; S, orange yellow; N, blue; Br, tawny. Hydrogen atoms omitted for clarity).

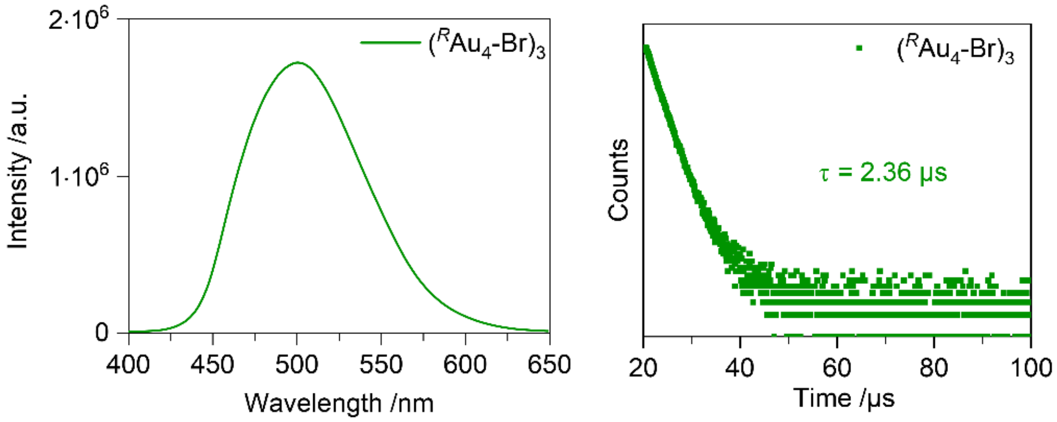

Figure S47. The emission spectrum and emission lifetime of $(^{R}Au_4\text{-}Br)_3$ cluster crystals measured under 365 nm excitation.

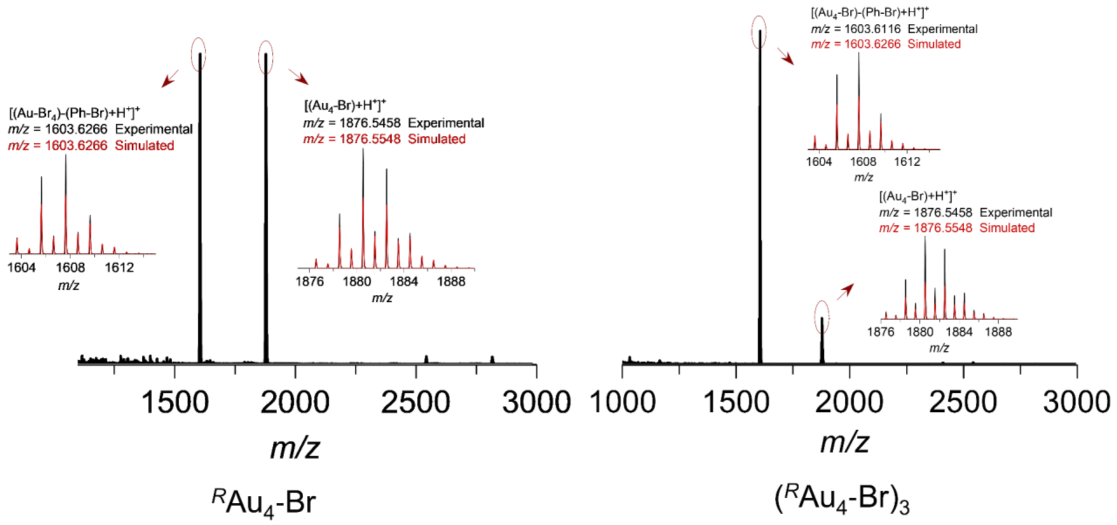

Figure S48. Positive mode ESI-MS of $^{R}Au_4$-Br and ($^{R}Au_4$-Br)$_3$ dissolved in DMF/CH_3CN. Insets: Enlarged portion of the ESI-MS exhibiting the measured (black line) and simulated (red line) isotopic distribution patterns in the *m*/*z* range of 1000-3000 with a charge state of +1.

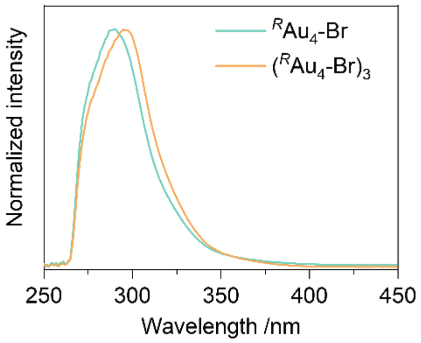

Figure S49. UV-Vis spectra of $^{R}Au_4$-Br (1.5×10^{-4} mol/L) and ($^{R}Au_4$-Br)$_3$ (5×10^{-5} mol/L) in DMF.

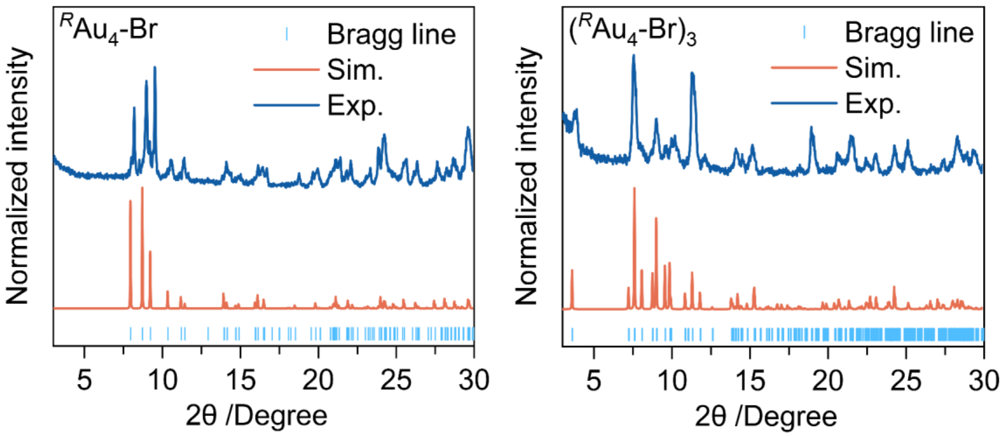

Figure S50 Powder XRD patterns of ^{R}Au$_4$-Br and (^{R}Au$_4$-Br)$_3$.

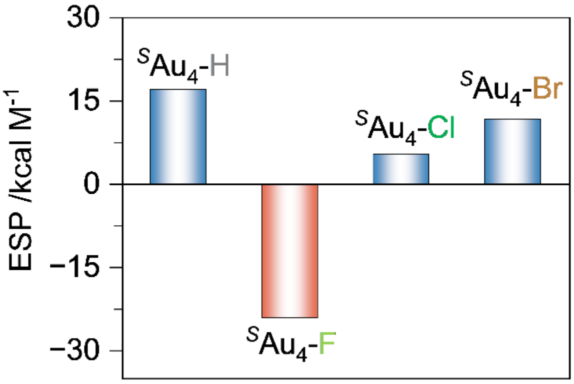

Figure S51. ESP of ^{S}Au$_4$-H and ^{S}Au$_4$-X (X = F, Cl, Br).

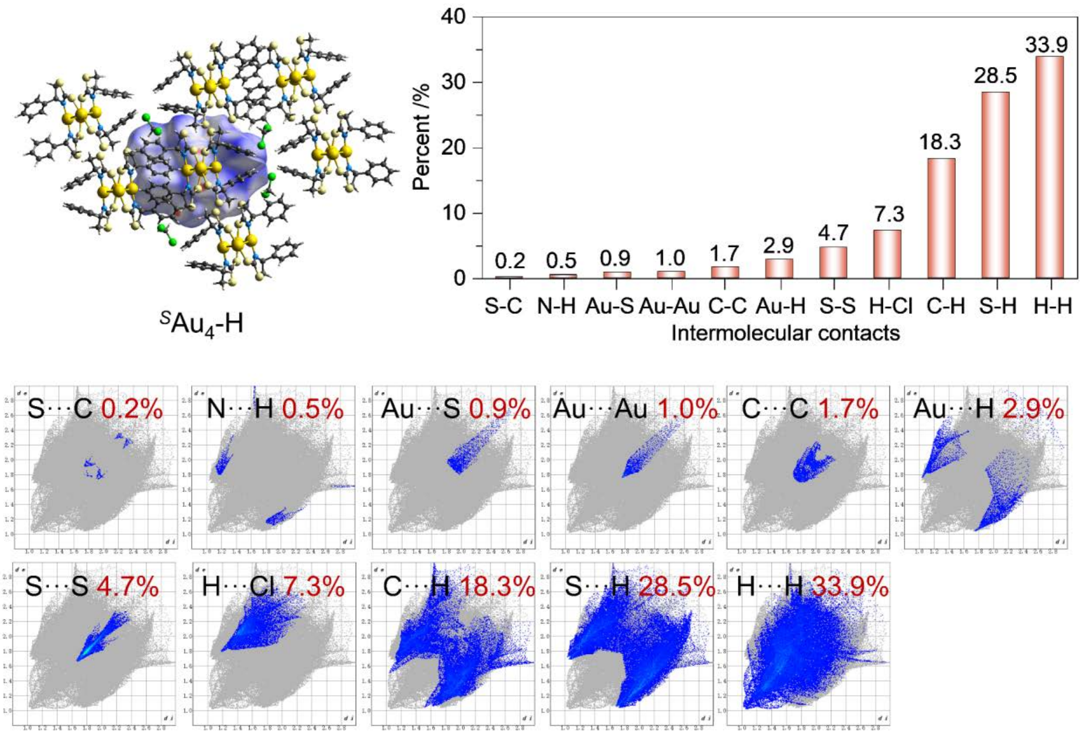

Figure S52. Hirshfeld surfaces of $^{S}Au_4$-H, percentage contributions of selected close intermolecular contacts based on the Hirshfeld surface area and two-dimensional fingerprint plots of $^{S}Au_4$-H.

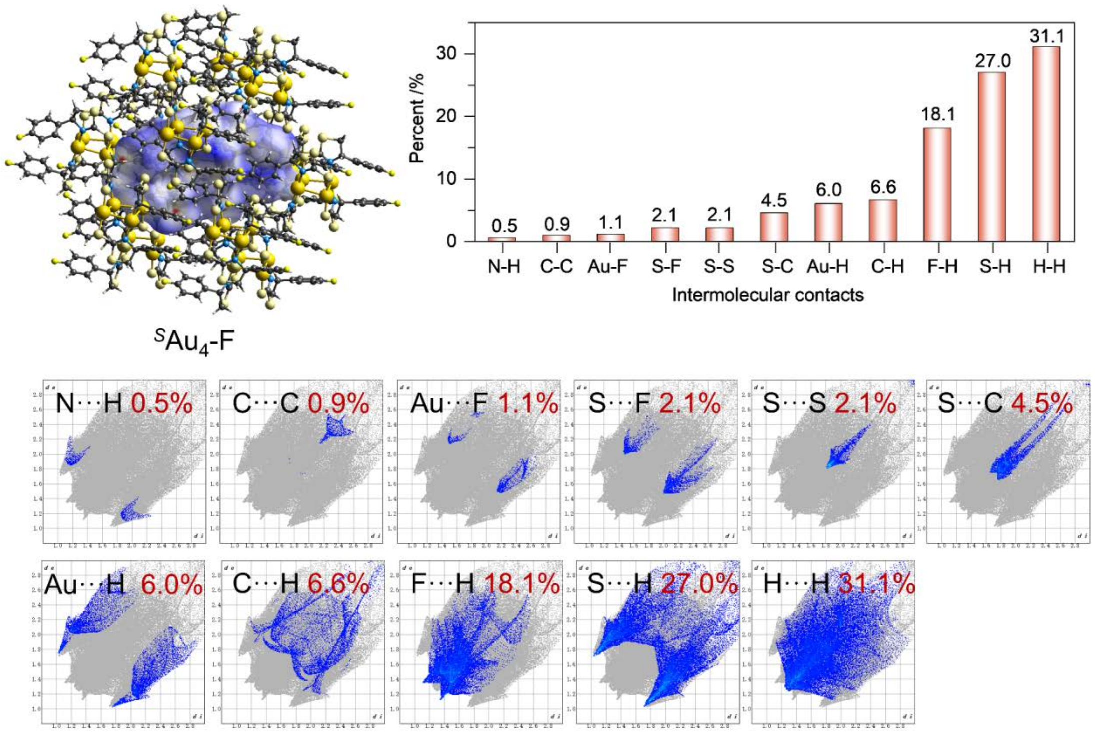

Figure S53. Hirshfeld surfaces of $^{S}Au_4$-F, percentage contributions of selected close intermolecular contacts based on the Hirshfeld surface area and two-dimensional

fingerprint plots of $^{S}Au_4$-F.

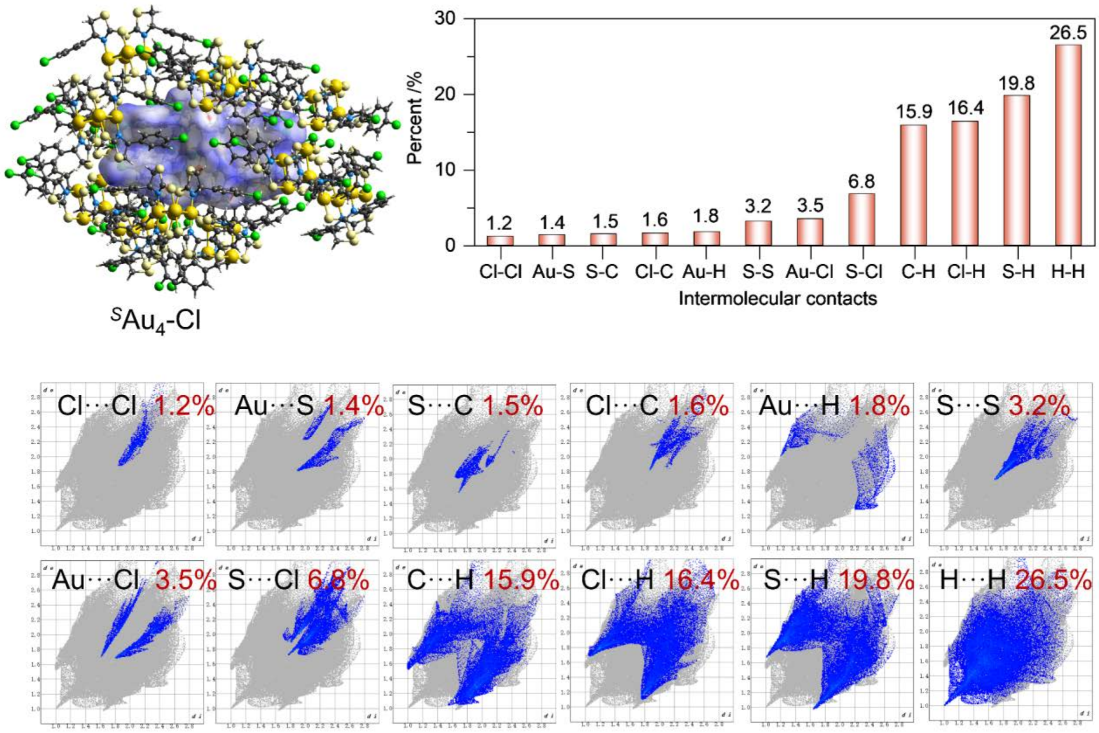

Figure S54. Hirshfeld surfaces of $^{S}Au_4$-Cl, percentage contributions of selected close intermolecular contacts based on the Hirshfeld surface area and two-dimensional fingerprint plots of $^{S}Au_4$-Cl.

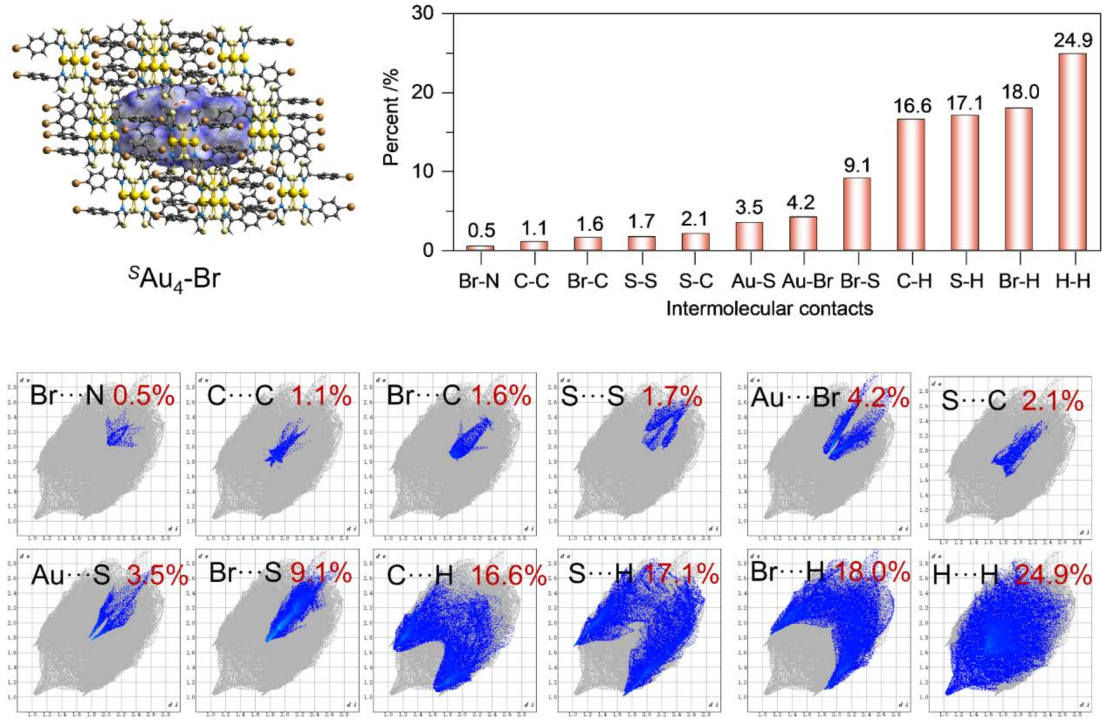

Figure S55. Hirshfeld surfaces of $^{S}Au_4$-Br, percentage contributions of selected close

intermolecular contacts based on the Hirshfeld surface area and two-dimensional fingerprint plots of $^{S}Au_4$-Br.

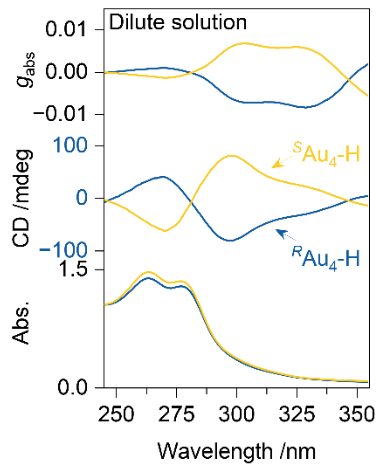

Figure S56. CD spectra of $^{R/S}Au_4$-H recorded in dichloromethane at 0.036 mM.

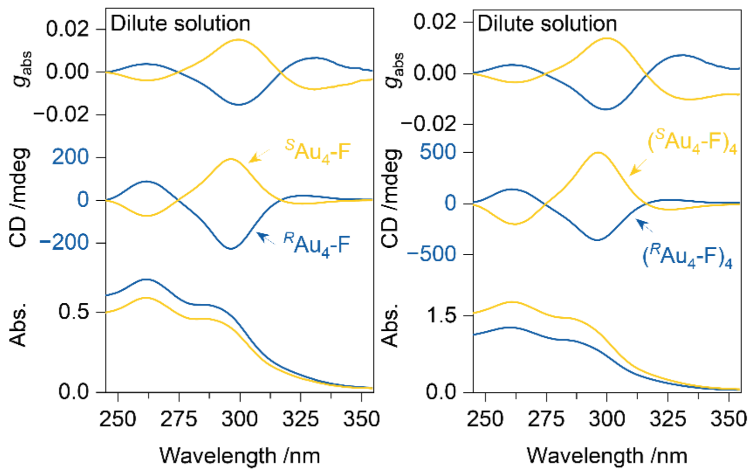

Figure S57. CD spectra of $^{R/S}Au_4$-F and $(^{R/S}Au_4$-F$)_4$ recorded in dichloromethane at 0.036 mM.

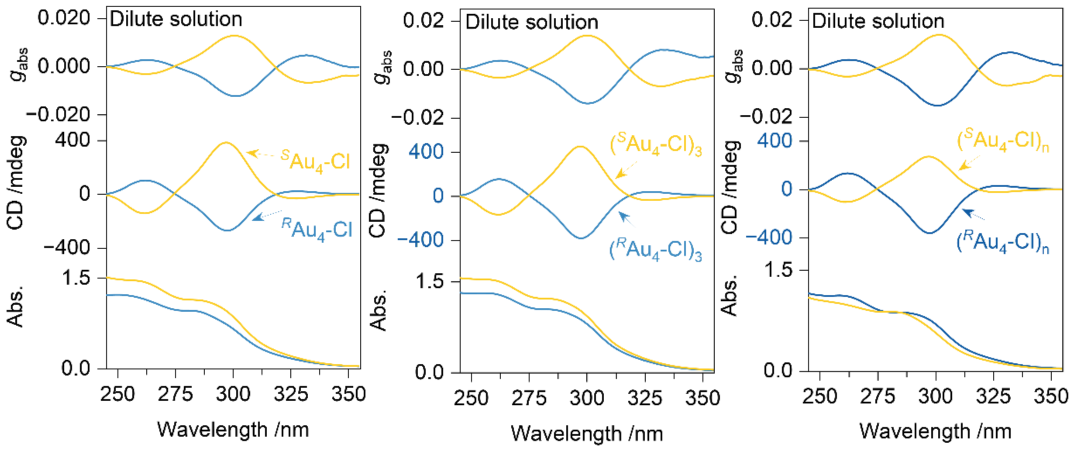

Figure S58. CD spectra of $^{R/S}$Au$_4$-Cl, ($^{R/S}$Au$_4$-Cl)$_3$ and ($^{R/S}$Au$_4$-Cl)$_n$ recorded in dichloromethane at 0.036 mM.

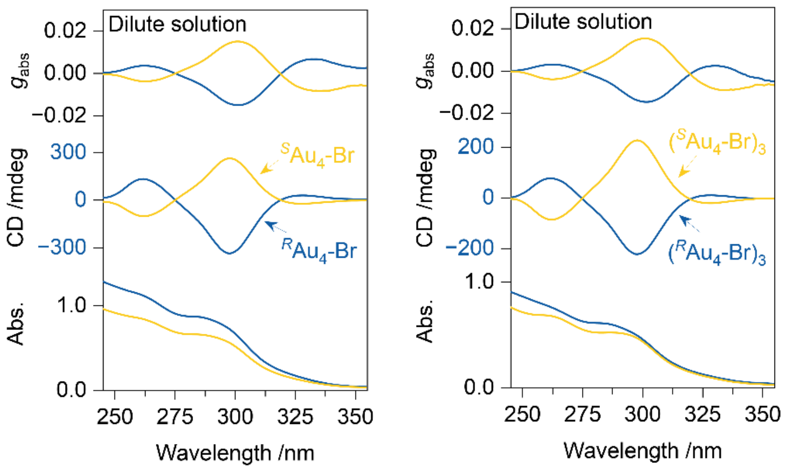

Figure S59. CD spectra of $^{R/S}$Au$_4$-Br and ($^{R/S}$Au$_4$-Br)$_3$ recorded in dichloromethane at 0.036 mM.

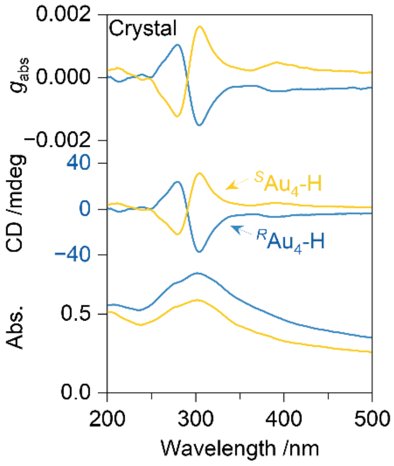

Figure S60. CD spectra of $^{R/S}$Au$_4$-H crystals.

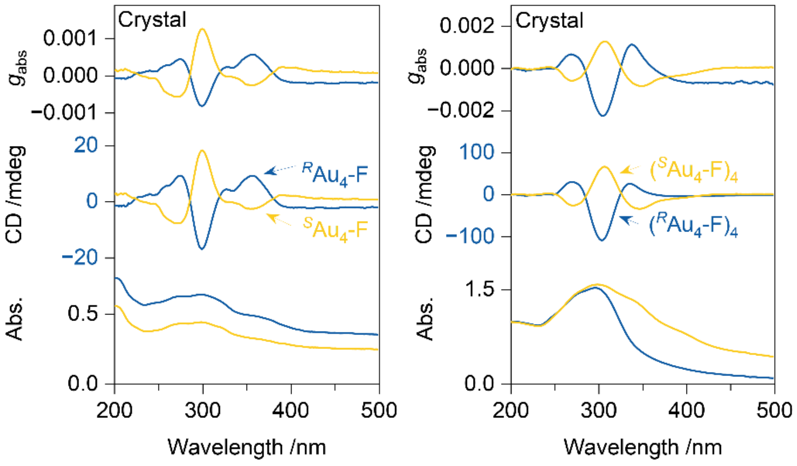

Figure S61. CD spectra of $^{R/S}$Au$_4$-F and ($^{R/S}$Au$_4$-F)$_4$ crystals.

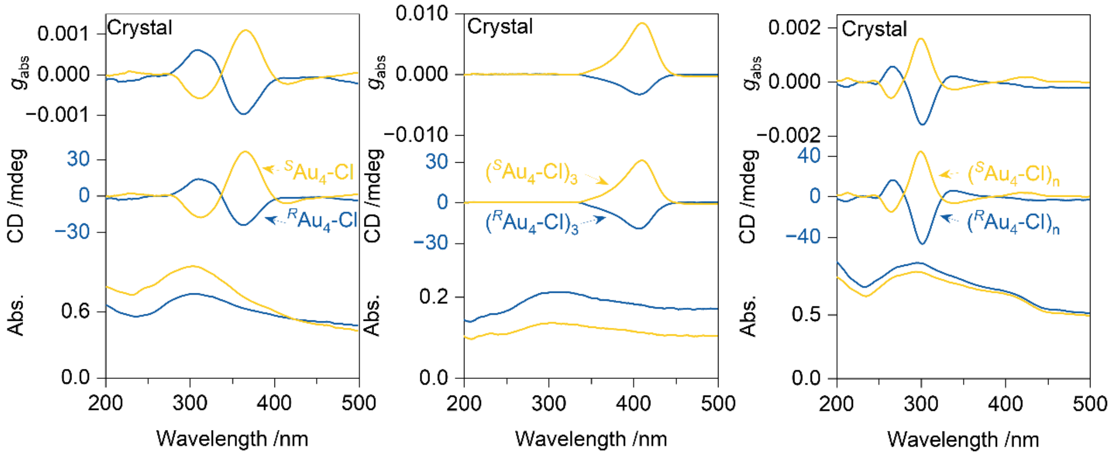

Figure S62. CD spectra of $^{R/S}$Au$_4$-Cl, ($^{R/S}$Au$_4$-Cl)$_3$ and ($^{R/S}$Au$_4$-Cl)$_n$ crystals.

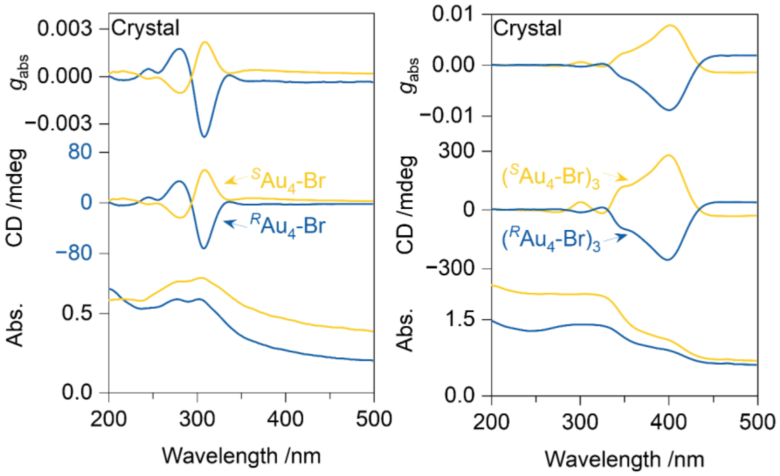

Figure S63. CD spectra of $^{R/S}$Au$_4$-Br and ($^{R/S}$Au$_4$-Br)$_3$ crystals.

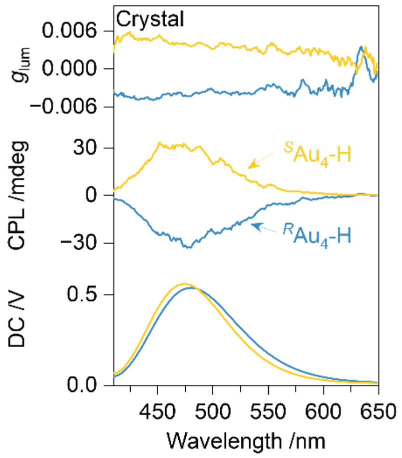

Figure S64. CPL spectra of $^{R/S}$Au$_4$-H crystals.

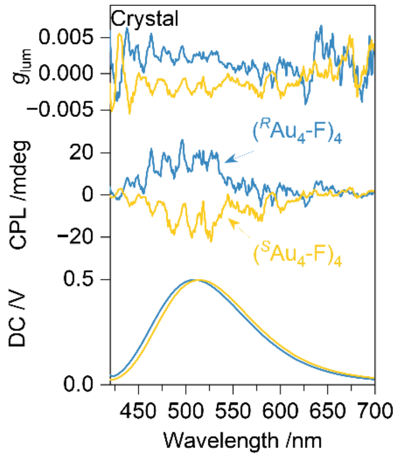

Figure S65. CPL spectra of ($^{R/S}$Au$_4$-F)$_4$ crystals.

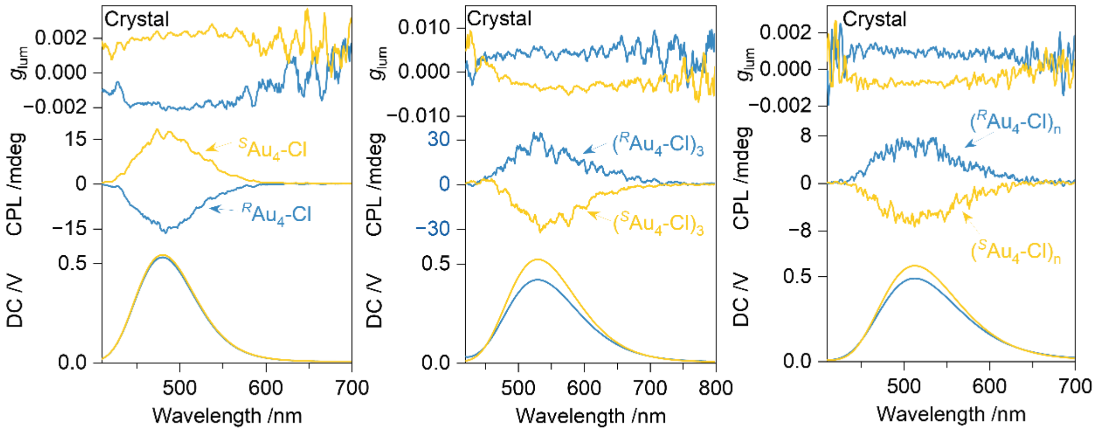

Figure S66. CPL spectra of $^{R/S}$Au$_4$-Cl, ($^{R/S}$Au$_4$-Cl)$_3$ and ($^{R/S}$Au$_4$-Cl)$_n$ crystals.

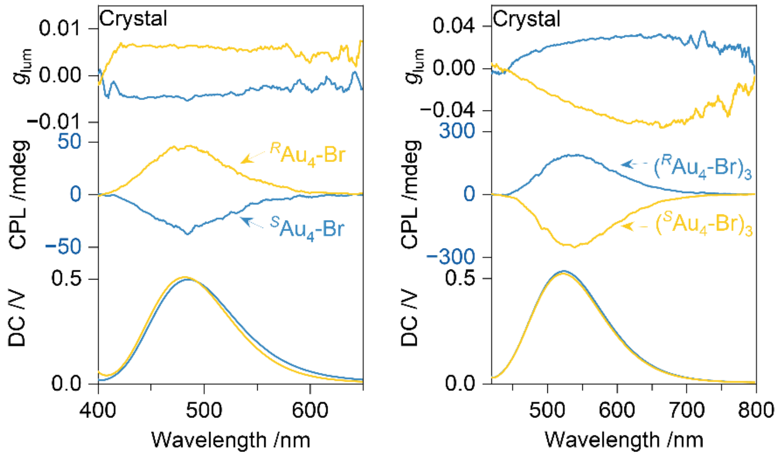

Figure S67. CPL spectra of $^{R/S}$Au$_4$-Br and ($^{R/S}$Au$_4$-Br)$_3$ crystals.

Table S1

Crystal data and structure refinement for ${}^{R}Au_4$-F and ${}^{S}Au_4$-F at 200 K.

	${}^{R}Au_4$-F	${}^{S}Au_4$-F
CCDC number	2455952	2455553
Identification code	${}^{R}Au_4$-F	${}^{S}Au_4$-F
Empirical formula	$C_{36}H_{28}Au_4F_4N_4S_8$	$C_{36}H_{28}Au_4F_4N_4S_8$
Formula weight	1635.87	1635.87
Temperature/K	200	200
Crystal system	orthorhombic	orthorhombic
Space group	$C222_1$	$C222_1$
a /Å	11.35250(10)	11.35240(10)
b /Å	19.0669(2)	19.07240(10)
c /Å	19.5353(2)	19.5289(2)
α /°	90	90
β /°	90	90
γ /°	90	90
Volume/ Å^3	4228.55(7)	4228.35(6)
Z	4	4
ρ_{calc} g/cm^3	2.571	2.571
μ/ mm^{-1}	29.637	29.638
F(000)	3008.0	3008.0
Crystal size/ mm^3	0.1 × 0.1 × 0.1	0.1 × 0.1 × 0.1
Radiation	Cu$K\alpha$ (λ = 1.54184)	Cu$K\alpha$ (λ = 1.54184)
2θ range for data collection/°	9.054 to 149.796	9.054 to150.436
Index ranges	$-14 \le h \le 14$, $-23 \le k \le 21$, $-24 \le l \le 24$	$-13 \le h \le 14$, $-23 \le k \le 21$, $-24 \le l \le 23$
Reflections collected	49953	22959
Independent reflections	4307 [R_{int} = 0.0835, R_{sigma} = 0.0269]	4310 [R_{int} = 0.0588, R_{sigma} = 0.0270]
Data/restraints/parameters	4307/18/253	4310/0/253
Goodness-of-fit on F^2	1.181	1.159
Final R indexes [I>=2σ (I)]	R_1 = 0.0327, wR_2 = 0.0895	R_1 = 0.0354, wR_2 = 0.0894
Final R indexes [all data]	R_1 = 0.0327, wR_2 = 0.0895	R_1 = 0.0355, wR_2 = 0.0894
Largest diff. peak/hole / e Å^{-3}	1.68/-3.53	1.45/-4.40
Flack parameter	-0.004(6)	-0.012(7)

[a] $R_1 = \Sigma||F_o| - |F_c||/\Sigma|F_o|$; [b] $wR_2 = \Sigma[w(F_o^2 - F_c^2)^2]/\Sigma[w(F_o^2)^2]^{1/2}$.

Table S2

Crystal data and structure refinement for (^{R}Au$_4$-F)$_4$ and (^{S}Au$_4$-F)$_4$ at 200 K.

	(^{R}Au$_4$-F)$_4$	(^{S}Au$_4$-F)$_4$
CCDC number	2455578	2455579
Identification code	(^{R}Au$_4$-F)$_4$	(^{S}Au$_4$-F)$_4$
Empirical formula	$(C_{36}H_{28}Au_4F_4N_4S_8)_4$	$(C_{36}H_{28}Au_4F_4N_4S_8)_4$
Formula weight	6543.48	6543.48
Temperature/K	200	200
Crystal system	monoclinic	monoclinic
Space group	I_2	C$_2$
a /Å	32.5905(2)	44.2014(7)
b /Å	13.9951(2)	14.0588(2)
c /Å	44.3459(4)	32.4865(4)
α /°	90	90
β /°	111.4300(10)	111.173(2)
γ /°	90	90
Volume/ Å^3	18828.1(4)	18824.9(5)
Z	16	16
ρ_{calc} g/cm^3	2.400	2.310
μ/ mm^{-1}	27.425	26.628
F(000)	12537.0	12032.0
Crystal size/ mm^3	0.4 × 0.22 × 0.19	0.3 × 0.1 × 0.1
Radiation	Cu$K\alpha$ (λ = 1.54184)	Cu$K\alpha$ (λ = 1.54184)
2θ range for data collection/°	5.826 to 147.488	4.226 to 157.964
Index ranges	$-39 \le h \le 40$, $-17 \le k \le 17$, $-48 \le l \le 54$	$-53 \le h \le 53$, $-17 \le k \le 17$, $-40 \le l \le 30$
Reflections collected	58821	128510
Independent reflections	26595 [R_{int} = 0.0422, R_{sigma} = 0.0565]	36836 [R_{int} = 0.1517, R_{sigma} = 0.1032]
Data/restraints/parameters	26595/2154/2592	36836/3880/2356
Goodness-of-fit on F^2	1.032	1.031
Final R indexes [I>=2σ (I)]	R_1 = 0.0503, wR_2 = 0.1335	R_1 = 0.0765, wR_2 = 0.2008
Final R indexes [all data]	R_1 = 0.0550, wR_2 = 0.1370	R_1 = 0.1009, wR_2 = 0.2204
Largest diff. peak/hole / e Å^{-3}	2.96/-1.50	2.08/-2.52
Flack parameter	0.040(8)	0.024(11)

^{a} $R_1 = \Sigma||F_o| - |F_c||/\Sigma|F_o|$; ^{b} $wR_2 = \Sigma[w(F_o^2 - F_c^2)^2]/\Sigma[w(F_o^2)^2]^{1/2}$.

Table S3

Crystal data and structure refinement for $^{R}Au_4$-Cl and $^{S}Au_4$-Cl at 200 K.

	$^{R}Au_4$-Cl	$^{S}Au_4$-Cl
CCDC number	2455574	2455575
Identification code	$^{R}Au_4$-Cl	$^{S}Au_4$-Cl
Empirical formula	$C_{36}H_{28}Au_4Cl_4N_4S_8$	$C_{36}H_{28}Au_4Cl_4N_4S_8$
Formula weight	1699.75	1699.75
Temperature/K	200	200
Crystal system	orthorhombic	orthorhombic
Space group	$P2_12_12_1$	$P2_12_12_1$
a /Å	35.1401(2)	35.13213(16)
b /Å	25.09600(10)	25.08870(11)
c /Å	10.03760(10)	10.04041(4)
α /°	90	90
β /°	90	90
γ /°	90	90
Volume/ Å^3	8851.92(11)	8849.81(7)
Z	8	8
ρ_{calc} g/cm^3	2.584	2.588
μ/ mm^{-1}	30.674	30.682
F(000)	6340.0	6356.0
Crystal size/ mm^3	0.2 × 0.1 × 0.1	0.2 × 0.1 × 0.1
Radiation	Cu$K\alpha$ (λ = 1.54184)	Cu$K\alpha$ (λ = 1.54184)
2θ range for data collection/°	6.14 to 147.729	4.328 to 147.63
Index ranges	$-43 \leq h \leq 43$, $-31 \leq k \leq 30$, $-12 \leq l \leq 7$	$-43 \leq h \leq 39$, $-30 \leq k \leq 31$, $-12 \leq l \leq 12$
Reflections collected	90549	99094
Independent reflections	17721 [R_{int} = 0.0827, R_{sigma} = 0.0620]	17757 [R_{int} = 0.0663, R_{sigma} = 0.0382]
Data/restraints/parameters	17721/6/1023	17757/0/1023
Goodness-of-fit on F^2	1.003	0.921
Final R indexes [I>=2σ (I)]	R_1 = 0.0420, wR_2 = 0.0770	R_1 = 0.0243, wR_2 = 0.0600
Final R indexes [all data]	R_1 = 0.0508, wR_2 = 0.0787	R_1 = 0.0258, wR_2 = 0.0605
Largest diff. peak/hole / e Å^{-3}	1.62/-2.06	1.36/-1.03
Flack parameter	-0.032(6)	-0.031(4)

^{a} $R_1 = \Sigma||F_o| - |F_c||/\Sigma|F_o|$; ^{b} $wR_2 = \Sigma[w(F_o^2 - F_c^2)^2]/\Sigma[w(F_o^2)^2]^{1/2}$.

Table S4

Crystal data and structure refinement for (^{R}Au$_4$-Cl)$_3$ and (^{S}Au$_4$-Cl)$_3$ at 200 K.

	(^{R}Au$_4$-Cl)$_3$	(^{S}Au$_4$-Cl)$_3$
CCDC number	2494681	2494812
Identification code	(^{R}Au$_4$-Cl)$_3$	(^{S}Au$_4$-Cl)$_3$
Empirical formula	$(C_{36}H_{28}Au_4Cl_4N_4S_8)_3$	$(C_{36}H_{28}Au_4Cl_4N_4S_8)_3$
Formula weight	5099.25	5099.25
Temperature/K	200	200
Crystal system	Monoclinic	Monoclinic
Space group	*C*2	*C*2
a /Å	23.1361(6)	23.1816(3)
b /Å	10.3710(3)	10.33350(10)
c /Å	24.0623(7)	23.9998(3)
α /°	90	90
β /°	99.034(3)	99.1380(10)
γ /°	90	90
Volume/ Å^3	5702.0(3)	5676.12(12)
Z	5	4
ρ_{calc} g/cm^3	2.484	2.491
μ/ mm^{-1}	29.490	29.621
F(000)	3924.0	3920.0
Crystal size/ mm^3	0.1 × 0.1 × 0.1	0.1× 0.1 × 0.1
Radiation	Cu*Kα* (λ = 1.54184)	Cu*Kα* (λ = 1.54184)
2θ range for data collection/°	7.44 to 148.36	7.462 to 148.032
Index ranges	$-21 \le h \le 28$, $-12 \le k \le 12$, $-28 \le l \le 30$	$-28 \le h \le 28$, $-12 \le k \le 12$, $-29 \le l \le 29$
Reflections collected	23336	61797
Independent reflections	9835 [R_{int} = 0.0845, R_{sigma} = 0.0800]	10949 [R_{int} = 0.0961, R_{sigma} = 0.0409]
Data/restraints/parameters	9835/169/683	10949/409/656
Goodness-of-fit on F^2	1.076	1.052
Final *R* indexes [I>=2σ (I)]	R_1 = 0.0752, wR_2 = 0.2007	R_1 = 0.0553, wR_2 = 0.1534
Final *R* indexes [all data]	R_1 = 0.0891, wR_2 = 0.2057	R_1 = 0.0571, wR_2 = 0.1555
Largest diff. peak/hole / e Å^{-3}	2.15/-1.66	3.40/-1.27
Flack parameter	-0.02(4)	-0.033(16)

[a] $R_1 = \Sigma||F_o| - |F_c||/\Sigma|F_o|$; [b] $wR_2 = \Sigma[w(F_o^2 - F_c^2)^2]/\Sigma[w(F_o^2)^2]^{1/2}$.

Table S5

Crystal data and structure refinement for ($^{R}Au_4$-Cl)$_n$ and ($^{S}Au_4$-Cl)$_n$ at 200 K.

	($^{R}Au_4$-Cl)$_n$	($^{S}Au_4$-Cl)$_n$
CCDC number	2455580	2455581
Identification code	($^{R}Au_4$-Cl)$_n$	($^{S}Au_4$-Cl)$_n$
Empirical formula	$C_{72}H_{56}Au_8Cl_8N_8S_{16}$	$C_{72}H_{56}Au_8Cl_8N_8S_{16}$
Formula weight	3399.5	3399.5
Temperature/K	200	200
Crystal system	orthorhombic	orthorhombic
Space group	$P2_12_12_1$	$P2_12_12_1$
a /Å	17.67260(10)	17.66720(10)
b /Å	17.85840(10)	17.86270(10)
c /Å	30.98550(10)	31.0036(2)
α /°	90	90
β /°	90	90
γ /°	90	90
Volume/ Å^3	9779.16(8)	9784.24(10)
Z	8	8
ρ_{calc} g/cm^3	2.275	2.274
μ/ mm^{-1}	27.507	27.493
F(000)	6049.0	6049.0
Crystal size/ mm^3	0.4 × 0.1 × 0.1	0.4 × 0.1 × 0.1
Radiation	Cu*Kα* (*λ* = 1.54184)	Cu*Kα* (*λ* = 1.54184)
2θ range for data collection/°	5.704 to 149.946	5.702 to 150.24
Index ranges	-22≤ *h* ≤ 22, -22 ≤ *k* ≤ 22, -38 ≤ *l* ≤ 38	-22≤ *h* ≤ 22, -22 ≤ *k* ≤22, -38≤ *l* ≤ 37
Reflections collected	258746	239359
Independent reflections	19951[R_{int} = 0.0870, R_{sigma} = 0.0273]	19904 [R_{int} = 0.0994, R_{sigma} = 0.0350]
Data/restraints/parameters	19951/0/1009	19904/0/1010
Goodness-of-fit on F^2	1.016	1.078
Final *R* indexes [*I*>=2*σ* (*I*)]	R_1 = 0.0405, wR_2 = 0.1155	R_1 = 0.0454, wR_2 = 0.1259
Final *R* indexes [all data]	R_1 = 0.0413, wR_2 = 0.1163	R_1 = 0.0520, wR_2 = 0.1449
Largest diff. peak/hole / e Å^{-3}	2.90/-3.52	3.20/-1.86
Flack parameter	-0.031(4)	-0.041(6)

[a] $R_1 = \Sigma||F_o| - |F_c||/\Sigma|F_o|$; [b] $wR_2 = \Sigma[w(F_o^2 - F_c^2)^2]/\Sigma[w(F_o^2)^2]^{1/2}$.

Table S6

Crystal data and structure refinement for ^{R}Au$_4$-Br and ^{S}Au$_4$-Br at 200 K.

	^{R}Au$_4$-Br	^{S}Au$_4$-Br
CCDC number	2455576	2455577
Identification code	^{R}Au$_4$-Br	^{S}Au$_4$-Br
Empirical formula	$C_{36}H_{28}Au_4Br_4N_4S_8$	$C_{36}H_{28}Au_4Br_4N_4S_8$
Formula weight	1875.55	1875.55
Temperature/K	200	219
Crystal system	triclinic	triclinic
Space group	P_1	P_1
a /Å	9.10752(18)	9.10310(10)
b /Å	11.28811(20)	11.28210(10)
c /Å	12.7962(2)	12.79820(10)
α /°	64.2393(18)	64.2910(10)
β /°	70.3169(18)	70.3030(10)
γ /°	77.1100(16)	77.1720(10)
Volume/ Å^3	1110.99(4)	1110.53(2)
Z	1	1
ρ_{calc} g/cm^3	2.811	2.812
μ/ mm^{-1}	32.269	32.282
F(000)	856.0	856.0
Crystal size/ mm^3	0.1 × 0.1 × 0.1	0.1 × 0.1 × 0.1
Radiation	Cu$K\alpha$ (λ = 1.54184)	Cu$K\alpha$ (λ = 1.54184)
2θ range for data collection/°	7.976 to 149.818	7.972 to 154.838
Index ranges	$-11\leq h\leq 11, -14\leq k\leq 13, -15\leq l\leq 16$	$-10\leq h\leq 11, -13\leq k\leq 14, -15\leq l\leq 15$
Reflections collected	20980	25498
Independent reflections	7936[R_{int} = 0.0472, R_{sigma} = 0.0382]	8159[R_{int} = 0.0225, R_{sigma} = 0.0189]
Data/restraints/parameters	7936/33/506	8159/3/505
Goodness-of-fit on F^2	1.035	1.069
Final R indexes [I>=2σ (I)]	R_1 = 0.0412, wR_2 = 0.1209	R_1 = 0.0179, wR_2 = 0.0469
Final R indexes [all data]	R_1 = 0.0446, wR_2 = 0.1253	R_1 = 0.0181, wR_2 = 0.0470
Largest diff. peak/hole / e Å^{-3}	1.99/-1.33	0.76/-1.11
Flack parameter	-0.02(2)	-0.024(5)

^{a} $R_1 = \Sigma||F_o| - |F_c||/\Sigma|F_o|$; ^{b} $wR_2 = \Sigma[w(F_o^2 - F_c^2)^2]/\Sigma[w(F_o^2)^2]^{1/2}$.

Reference

1. Han Z., Zhao X., Peng P., *et al.*, Intercluster aurophilicity-driven aggregation lighting circularly polarized luminescence of chiral gold clusters. *Nano Res* 2020; **13**: 3248-3252.

2. Sheldrick G., A short history of SHELX. Acta Crystallographica Section A 2008; **64**: 112-122.

3. Dolomanov O. V., Bourhis L. J., Gildea R. J., *et al.*, OLEX2: a complete structure solution, refinement and analysis program. *J Appl Crystallogr* 2009; **42**: 339-341.

4. Sheldrick G., SHELXT - Integrated space-group and crystal-structure determination. *Acta Crystallographica Section A* 2015; **71**: 3-8.

5. Hubschle C. B., Sheldrick G. M., Dittrich B., ShelXle: a Qt graphical user interface for SHELXL. *J Appl Crystallogr* 2011; **44**: 1281-1284.

6. Cheng S., Chen Z., Yin Y., *et al.*, Progress in mechanochromic luminescence of gold(I) complexes. *Chin Chem Lett* 2021; **32**: 3718-3732.

7. Huitorel B., Benito Q., Fargues A., *et al.*, Mechanochromic luminescence and liquid crystallinity of molecular copper clusters. *Chem Mater* 2016; **28**: 8190-8200.

8. Benito Q., Maurin I., Cheisson T., *et al.*, Mechanochromic luminescence of copper iodide clusters. Chemistry – *A European Journal* 2015; **21**: 5892-5897.

9. Wu J., Liu D., Lu Y.-B., *et al.*, Investigations on the strong light-changed

photoluminescence performance and the crystalline structure of $MAPbBr_1Cl_2$ Mixed Halide Perovskites. *The Journal of Physical Chemistry C* 2023; **127**: 23428-23435.

10. Baranov A. Y., Doronina E. P., Bagryanskaya I. Y., *et al.*, Brightly emissive octahedral Cu_4X_4 clusters showing polymorphic-dependent and mechanochromic phosphorescence. *Dalton Trans.* 2025; **54**: 7014-7022.

11. Luty-Błocho M., Cyndrowska J., Rutkowski B., *et al.* Synthesis of gold clusters and nanoparticles using cinnamon extract—a mechanism and kinetics study. *Molecules* 2024; **29**: 1426.

12. Hou B., Yang S., Li B., *et al.*, Construction of multi-hydroxyl/ketone lanthanide metal–organic frameworks for understanding mechanochromic luminescence and high proton conductivity. *Inorg Chem Front* 2022; **9**: 4376-4384.
